# Flexible parametric methods for calculating life expectancy in small populations

**DOI:** 10.1186/s12963-023-00313-x

**Published:** 2023-09-13

**Authors:** Freya Tyrer, Yogini V. Chudasama, Paul C. Lambert, Mark J. Rutherford

**Affiliations:** 1 Biostatistics Research Group, Department of Population Health Sciences, George Davies Centre, University of Leicester, University Road, Leicester, LE1 7RH UK; 2 Leicester Real World Evidence Unit, Diabetes Research Centre, University of Leicester, Leicester, UK; 3 Department of Medical Epidemiology and Biostatistics, Karolinska Institutet, Stockholm, Sweden

**Keywords:** Life expectancy, Epidemiology, Flexible parametric methods, Electronic health records, Chiang, Observational studies

## Abstract

**Background:**

Life expectancy is a simple measure of assessing health differences between two or more populations but current life expectancy calculations are not reliable for small populations. A potential solution to this is to borrow strength from larger populations from the same source, but this has not formally been investigated.

**Methods:**

Using data on 451,222 individuals from the Clinical Practice Research Datalink on the presence/absence of intellectual disability and type 2 diabetes mellitus, we compared stratified and combined flexible parametric models, and Chiang’s methods, for calculating life expectancy. Confidence intervals were calculated using the Delta method, Chiang’s adjusted life table approach and bootstrapping.

**Results:**

The flexible parametric models allowed calculation of life expectancy by exact age and beyond traditional life expectancy age thresholds. The combined model that fit age interaction effects as a spline term provided less bias and greater statistical precision for small covariate subgroups by borrowing strength from the larger subgroups. However, careful consideration of the distribution of events in the smallest group was needed.

**Conclusions:**

Life expectancy is a simple measure to compare health differences between populations. The use of combined flexible parametric methods to calculate life expectancy in small samples has shown promising results by allowing life expectancy to be modelled by exact age, greater statistical precision, less bias and prediction of different covariate patterns without stratification. We recommend further investigation of their application for both policymakers and researchers.

**Supplementary Information:**

The online version contains supplementary material available at 10.1186/s12963-023-00313-x.

## Background

Government and policymakers across the world are committed to reducing health disparities in the most vulnerable, including people living in deprived areas, the elderly and people with disabilities [1, 2]. A key indicator of health is mortality, and it is often advantageous to use life expectancy to compare differences in survival between populations.

One of the limitations of many of the methods used to calculate life expectancy is that they are known to be inaccurate for small populations. For example, the Chiang’s abridged life table approach [3, 4] used by the Office for National Statistics in the UK [5], and similar approaches, such as those proposed by Silcocks and colleagues [6], require the relevant sub-populations to be stratified before comparisons can be made. Because of this, sub-populations with fewer than 5000 person-years are not recommended [7]. These methods also require splitting ages into groups (usually 5-year intervals [7]) and creating an open-ended final age group (e.g. 80+ years) in which one death has to occur for the life expectancy calculation to work. Where numbers are small or in sub-populations that are vulnerable to premature mortality, this final age group may need to start relatively young thereby assuming similar risk profiles across older ages in the comparison population, which may not be sensible. While other approaches, such as Bayesian random effects models to extrapolate information from different areas/regions, have been proposed as a way of dealing with small populations [8], they have not been routinely adopted by researchers or policymakers.

For this work, we investigated the use of flexible parametric methods to calculate life expectancy in small populations. We described and compared life expectancy using individual-level censored data from a real-life case study from the Clinical Practice Research Datalink (CPRD) and linked mortality data for the general population and people with intellectual disabilities, stratified by Type 2 diabetes mellitus (T2DM) status.

## Methods

### Overview of survival analyses and flexible parametric methods

A glossary of terms for this work is provided in Additional File 1 (Table S1). Survival, or time-to-event, analyses are commonly used in epidemiological research because they consider the time it takes to develop a given event. This is important to establish whether some groups of individuals die earlier than others. Nearly all survival approaches involve analysing survival in the presence of censored data [9] whereby individuals are (right) censored if they do not have the event but can no longer be followed up.

Depending on the research question of interest, different functions are used to model the data distribution in time-to-event analyses. Perhaps the most common of these is the hazard function, $h\left( t \right)$, which is defined as the rate of failure between $t$ and $\Delta t$ (i.e. a miniscule time period; $\Delta \to 0$), conditional on the individual not experiencing the event of interest by time $t$. The cumulative hazard function, $H\left( t \right)$, is the integral of the hazard function, $h\left( t \right)$*,* over the entire distribution of t (i.e. time 0 and time $t$) for which the baseline formula is given below.

$$H_{0} \left( t \right) = \mathop \smallint \limits_{o}^{t} h_{0} \left( u \right){\text{d}}u$$

Another common function used in time-to-event analyses is the survival function, S(t), which is the probability that an individual will survive beyond time $S\left( t \right)$, $\Pr (T > t)$. It has a relationship with the cumulative hazard function, as below:

$$S\left( {t|{\varvec{x}}_{{\varvec{i}}} } \right) = {\text{exp}}( - {\text{H}}_{0} \left( t \right)\exp \left( {{\varvec{x}}_{{\varvec{i}}} {\varvec{\beta}}} \right) = \exp \left( { - H_{i} \left( t \right)} \right)$$

where $x_{i} \beta$ represents the linear predictor, $\beta$ the log hazard ratios and $x_{{\varvec{i}}}$ the covariate values for the *i*th individual.

There are three main approaches to analysing survival data: Non-parametric; semi-parametric (e.g. Cox proportional hazards model [10]); and parametric. Non-parametric and semi-parametric methods allow baseline hazard functions to vary freely, which means that they make no assumptions about the shape of the underlying hazard or death rate. Parametric methods specify a parametric form for the (cumulative) baseline hazard function, which can be useful for precision but they also come with constraints in terms of their ability to model fluctuations in the data over time, in particular turning points.

Flexible parametric methods, first introduced in 2002 [13] and extended further in 2009 [14], allow a parametric form to be specified for the baseline hazard function but also use restricted cubic splines to flexibly and smoothly capture the shape of the baseline log (cumulative) hazard function over time. The use of splines to estimate mortality rates is well-recognised in the actuarial and demography literature [11, 12] and is increasingly used by epidemiologists and statisticians [15]. For flexible parametric models, the spline function is defined by constrained cubic polynomial functions forced to join at a pre-selected number of joining points, called knots, which equate to the degree of complexity, often expressed as degrees of freedom. Knots are usually spaced equally as percentiles across the distribution of event times, and the splines are constrained to be linear beyond the boundary knots (i.e. at the extremes of the curve). For example, the default knot placement for flexible parametric methods in the statistical package Stata (‘*stpm2*’) [14, 16] for four degrees of freedom is at the 0th, 25th, 50th, 75th and 100th percentile of the event distribution for a specific population. In this study, we used age as the timescale of interest as this is a natural choice for life expectancy calculations and aligns with previous research in this area [17–20]. The knots were, therefore, placed according to the distributions of age at death in the study population.

### Calculation of life expectancy using flexible parametric methods

More details of the survival function for flexible parametric methods and Stata code (v16.0) for calculating life expectancy are shown in the supplementary material (Additional file 1: Box S1 and S2).

Additional years expected to live from a given age, $a$*,* to a maximum age, $w$*,* can be estimated fitting a flexible parametric model and integrating under the survival function curve (i.e. calculating the area under the curve) to age $w$, scaled by the survival function (i.e. proportion live) at age $a$ using the formula below.

$$\mathop \int \limits_{a}^{w} \frac{{S\left( t \right){\text{d}}t}}{S\left( a \right)}$$

This can be approximated using numerical integration techniques in statistical software.

**Fig. 1:**
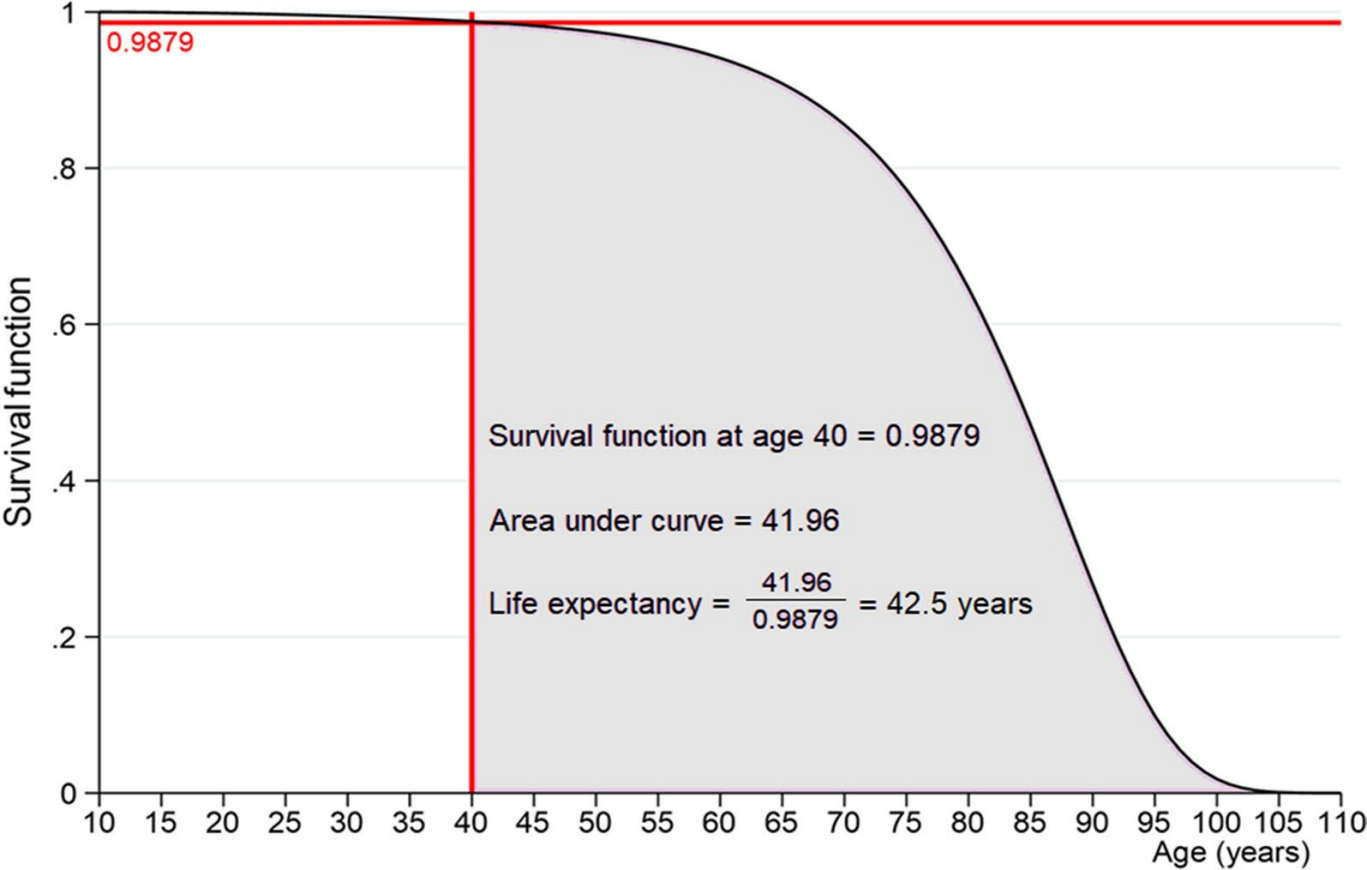
Illustration of how average life expectancy in people aged 40 years is calculated from the survival function of a flexible parametric model

The example in Fig. 1 illustrates how life expectancy of people aged 40 years in a given population can be calculated using flexible parametric methods. The figure shows the survival function for all ages after fitting a flexible parametric model with 4 degrees of freedom using age as the time scale. We can see that the survival function at age 40 years, denoted by the horizontal red line, is 0.9879. Therefore, the additional years expected to live is the integral of the survival function from 40 to the maximum age ($w$ = 110 years), conditional on surviving to this age. The area under the curve is 41.96 years. Therefore, people aged 40 years in this population can expect, on average, to live for an additional 42.5 years and have an overall average life expectancy of 82.5 years.

A period life expectancy estimate is given in a similar way to Chiang’s methods [3, 4], by combining the current age-specific rates of mortality in the most recent calendar year to calculate life expectancy. Parametric models are particularly good for this as we are able to model a smooth representation across age, borrowing strength accordingly.

The theoretical advantages of flexible parametric methods over traditional life expectancy approaches include the modelling of life expectancy with greater statistical precision because fewer parameters are used. For example, taking 5-year-age groups from birth to 75+ years would require 16 parameters under the Chiang approach as compared with the five parameters described in the example. The methods also allow age to be modelled by exact age, which means that individual-level average risk can be estimated, and there are not the same age restrictions at the tail of the distribution because age is not truncated. Flexible parametric approaches also have the potential to allow prediction for different covariate patterns by modelling age-varying effects (i.e. allowing the proportional differences between the covariates to change across different ages) using interaction terms by the covariates and spline variables for age [21].

### Data sources

The case example for this work used individual-level censored data from the Clinical Practice Research Datalink (CPRD GOLD), linked (person-level) with hospital episode statistics (HES) and death registrations from the Office for National Statistics (approved study protocol number: 19_267RA3). The CPRD is an electronic health record research database of more than 11.3 million patients, broadly representative of the national population in terms of age, gender and ethnicity [22], from general practice (GP) surgeries in the UK. The study comprised GP surgeries in England only—of which approximately 75% consent to linkage to deaths data. Under the UK healthcare system, most individuals are registered with a general practitioner (primary care physician) and use them as the first point of contact for health problems.

The study followed the Reporting of studies Conducted using Observational Routinely‐collected health Data (RECORD) checklist [23] (see Additional file 1: Table S2). People with intellectual disabilities were identified from a pre-agreed set of primary care Read codes and has been described in previous research (see Additional file 1: Table S2) [24]. Diagnostic codes for Type 2 diabetes were identified using previous literature [25] and are described in Additional file 1: Table S3. The initial extract from the CPRD has been described previously [24] and was based on the following inclusion criteria: Registered at the GP surgery between 1 Jan 2000 and 29 Sept 2019, and 10 years old or over to account for delays in reporting of diagnoses of intellectual disability in children [26]. An additional 23 patients with Angelman or Cockayne syndrome were added in August 2021 after an amendment to the original protocol (approved March 2020 but delayed during the COVID period). A simple random sample of people without intellectual disabilities (initially 1 million from 2000 to 2019 before exclusions) was used for the comparison group with the same eligibility criteria (but without a diagnosis of intellectual disability).

### Statistical analyses

For the purposes of this work and to align with standard period life expectancy calculations, we restricted the observation time to a 1-year period (2012) (i.e. person-time only contributed to this calendar year). Date of entry into the cohort was defined as the latest date according to the person and GP surgery’s characteristics: 01 Jan 2012; date of registration with the GP surgery; date the GP surgery was defined as being up to standard for research purposes (using the CPRD’s own quality indicators); or date the individual turned 10 years old (to align with the eligibility criteria). Because there are known delays in reporting intellectual disability diagnoses [27] and to avoid conditioning on the future, intellectual disability status was treated as an age-dependent covariate such that people with intellectual disabilities contributed to the comparison cohort prior to their first diagnosis. T2DM status was also allowed to change during the observation period. Date of exit was defined as: Date of death; date of end of calendar period; date of last GP surgery update; date the individual left the GP surgery; or 31 Dec 2012, whichever was first. For T2DM, the baseline measure at 2012 was taken; people in the cohort who developed T2DM after 1 January 2012 were treated as being T2DM-free during the entire 1-year period.

Table 1 summarises the models compared. We estimated life expectancy and 95% confidence intervals by intellectual disability and T2DM status using: Fully stratified models (Method 1: Fully stratified); with intellectual disability stratified but T2DM as an age-varying covariate (Method 2: Partially stratified); and for the entire population with both T2DM and intellectual disability as age-varying covariates, also fitting an interaction term (Method 3: Full model). Follow-up started from adulthood (age 20+ years) because T2DM is known to be relatively uncommon in younger ages [28], and life expectancy was reported for people aged 40+ years only. The models were also compared with Chiang’s abridged life table approach, stratified by intellectual disability and T2DM status.

**Table 1:** Summary of flexible parametric methods used to estimate life expectancy in people with and without intellectual disabilities and T2DM

Name of method	Approach to analysis
Method 1: Fully stratified	Four individual models for: (a) Intellectual disabilities and T2DM (b) intellectual disabilities and no T2DM (c) no intellectual disabilities and T2DM (d) no intellectual disabilities and no T2DM
Method 2: Partially stratified	Two individual models for: (a) Intellectual disabilities (b) no intellectual disabilities Interaction between T2DM and age modelled
Method 3: Full model	One model of entire sample population Interaction between age and intellectual disability and T2DM modelled
Method 4: Full model with adapted knots	One model of entire sample population, but with knots forced to intellectual disability sample (i.e. minority group) Interaction between age and intellectual disability and T2DM modelled

For the flexible parametric models, life expectancy estimates and confidence intervals were calculated using the Delta method after fitting models with 4 degrees of freedom using ‘*stpm2*’ in Stata (v16.0) [16]. We chose not to use the default knot placements (0th, 25th, 50th, 75th and 100th percentile) for this work (with age as the timescale) owing to the sparsity of events at the tails of the distributions. Instead, we chose to use the knot placements ‘where data exist’ recommended by Harrell [29] at the 5th, 27.5th, 50th, 72.5th and 95th percentile. However, as a sensitivity analysis, we repeated the analyses using the default knots for the models. We also calculated life expectancy in the entire population after forcing knot placements to match the event distribution in the intellectual disability population (Methods 4: Full model with adapted knots)—see Table 1. The variance of the estimate of life expectancy was calculated on the log scale in order to stabilise the variance and avoid negative confidence intervals.

For older individuals with intellectual disabilities with/without T2DM, sample sizes could become very small. Therefore, confidence intervals were also compared with percentile-based bootstrapped confidence intervals in people aged 80–99 years. As an additional validation of the confidence intervals derived from the model, these were compared with both percentile-based and normal-based confidence intervals after bootstrapping in the larger sample without intellectual disabilities at the older ages (95–105 years).

For the Chiang’s abridged life table approach, confidence intervals were calculated using the adjusted Chiang approach advocated by Eayres and Williams [7], which involves adding a correction term to the original Chiang variance to incorporate length of survival in the last age group [30]. When calculating life expectancies in small populations, Chiang’s methods with the adjusted variance are recommended over Silcocks’ methods [7].

To assess the performance of the fully stratified FPM approach (Method 1) compared to the model borrowing strength from the larger covariate groupings (full model, Method 3), we conducted a small simulation study using the CPRD data as the basis for the data generation. In order to generate data across 1000 simulation replications, we fitted a stratified Gompertz model [31, 32] to each of the 4 groupings of covariate profile detailed in Table 1 (a–d) with age as the timescale and took these to be our true values. We generated the simulated survival times using the approach described by Bender et al*.* [33]. A multinomial logistic regression was fitted to the data to obtain estimates of the proportions in each of the 4 groupings and separate regression models for age at study entry in each of the 4 groupings using the approach described by Smith et al*.* [34] to recreate continuous covariate distributions through a regression with splines based on an inverse-normal-rank-based-transformation. The parameter estimates from these set of statistical models were used to repeatedly generate replications of the whole cohort with the same follow-up restrictions as the main study using the exact sample size observed in the original cohort (*N* = 453,091—including the 1871 individuals who changed T2DM status during the observation window). These replications, therefore, differed in covariate distribution (age and population subgroup) and survival time values/event indicator, but were based on the parameter estimates above, leading to the same degree of sparsity for group (a) [intellectual disability and T2DM] particularly.

We then fitted Method 1 and Method 3 to each of the 1000 simulated datasets to compare the stratified and full models against the true value for the life expectancy according to the known parameters for the Gompertz distribution. Only estimates where both models converged were compared. We focused on prediction for the smallest group (group a) at older ages because this group had the smallest sample size. As the simulation was based on the sample data for 2012, which may have been different from the true shape of the distributions in this CPRD study, we also repeated the simulation exercise in an extended sample 2 years either side to include data over a 5-year period from 1 January 2010 to 31 December 2014.

## Results

The population in 2012 comprised 16,904 individuals with intellectual disabilities (*n* = 1006 with T2DM) and 434,318 people without intellectual disabilities (*n* = 23,381 with T2DM). The characteristics of the study population are shown in Table 2.

**Table 2:** Characteristics of 2012 sample population used for the analysis

Characteristic	Intellectual disabilities *N* (%)/Median (range)		No intellectual disabilities *N* (%)/Median (range)	
Total	16,904	(100.0)	434,318^a^	(100.0)
Age (at baseline^b^)	36.0	(10–100)	44.0	(10–109)
Gender				
Male	9,451	(55.9)	213,678	(49.2)
Female	7,453	(44.1)	220,640	(50.8)
Ethnicity				
White	12,796	(75.7)	297,320	(68.5)
South Asian	459	(2.7)	15,425	(3.6)
Black	374	(2.2)	11,257	(2.6)
Other	456	(2.7)	16,428	(3.8)
Not known	2819	(16.7)	93,888	(21.6)
Type 2 diabetes^c^				
Present	1006	(6.0)	23,381	(5.4)
Died	193	(1.1)	4296	(1.0)
Most common genetic syndromes				
Down syndrome	2049	(12.1)		
Fragile X syndrome	323	(1.9)		
Tuberous sclerosis	158	(0.9)		
William syndrome	70	(0.4)		
Prader-Willi syndrome	61	(0.4)		

^a^Includes 553 individuals from the intellectual disability sample who changed status (i.e. had their first intellectual disability diagnosis) during the observation window and a further 1339 individuals who were registered but had their first intellectual disability diagnosis outside the window (i.e. after 2012)

^b^Individuals entered the cohort on 01/01/2012 or sometime during 2012 if registered or changed status during the observation period

^c^Includes 1871 individuals who changed T2DM status (i.e. had a T2DM diagnosis) during the observation window

### Life expectancy by intellectual disability and T2DM status

**Fig. 2:**
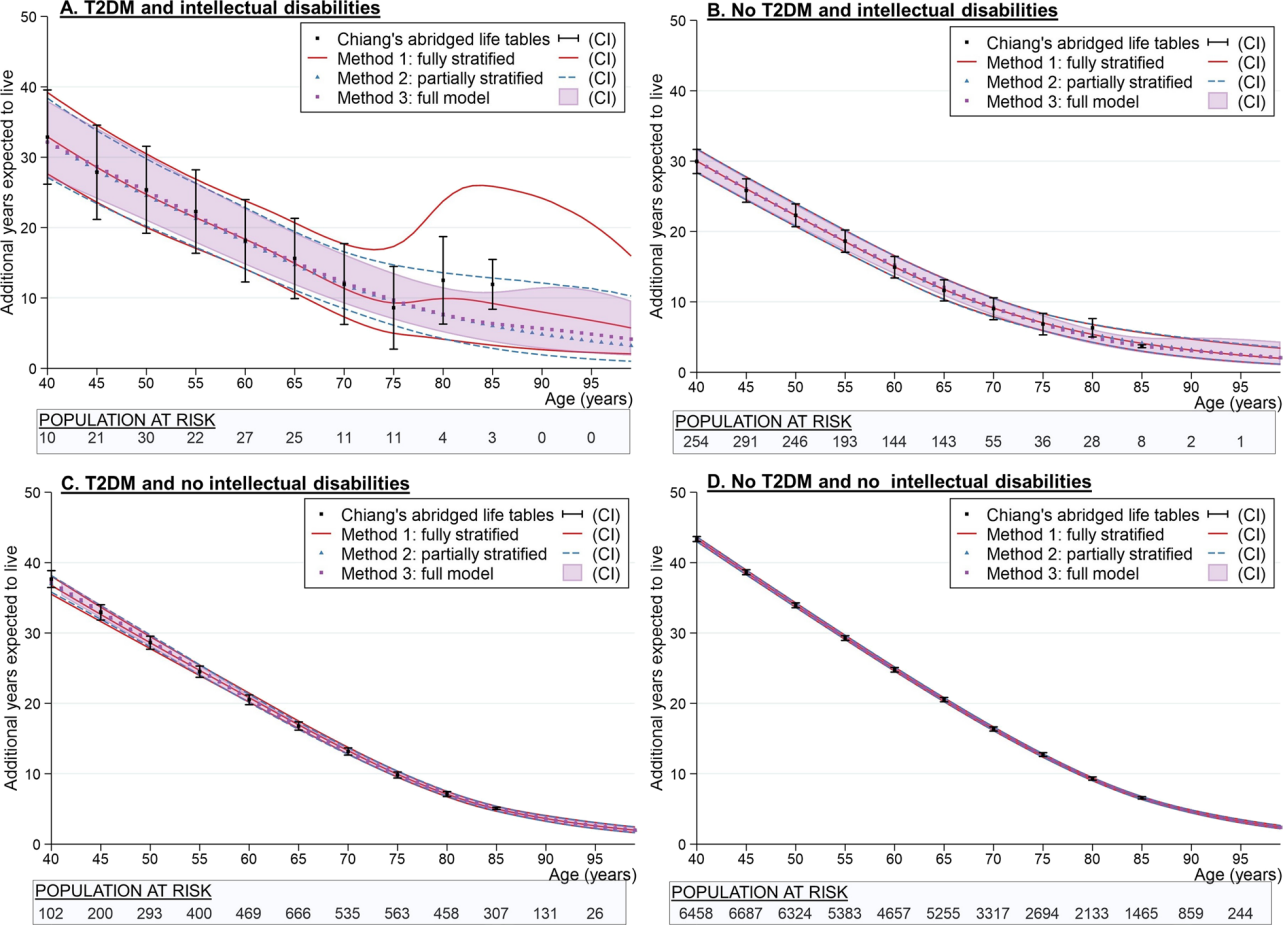
Comparison between flexible parametric methods^ab^ and Chiang’s abridged life table^cd^ for estimating life expectancy in small populations ID: Intellectual disability; T2DM: Type 2 diabetes mellitus; CI confidence interval; TVC = age-varying covariate knots. ^a^Knot placements (years): Method 1: Fully stratified: ID & T2DM: 36.8, 53.4, 64.5, 67.9, 75.1; ID & No T2DM: 24.4, 42.5, 55.0, 63.4, 82.3; No ID & T2DM: 50.5, 66.6, 72.8, 77.2, 84.9; No ID & No T2DM: 40.7, 64.8, 73.8, 79.5, 87.4]; Method 2: Partially stratified: ID: 27.0, 43.5, 55.3, 65.0, 81.8 [TVC 47.0, 63.7]; No ID: 42.8, 65.3, 73.5, 79.1, 87.0 [TVC 67.8, 77.9]]; Method 3: Full model: 40.7, 64.4, 73.0, 79.0, 86.9 [TVC 67.0, 77.5]]. ^b^Additional years expected to live from age 40 years (95% CI): Method 1: Fully stratified: ID & T2DM: 32.9 years (27.6, 39.2); ID & No T2DM: 30.0 years (28.4, 31.7); No ID & T2DM: 36.8 years (35.5, 38.1); No ID & No T2DM: 43.3 years (43.0, 43.7); Method 2: Partially stratified: ID & T2DM: 32.3 years (27.1, 38.4); ID & No T2DM: 30.0 years (28.4, 31.7); No ID & T2DM: 37.0 years (35.8, 38.2); No ID & No T2DM: 43.3 years (43.0, 43.6); Method 3: Full model: ID & T2DM: 32.1 years (27.1, 38.0); ID & No T2DM: 30.0 years (28.4, 31.7); No ID & T2DM: 37.2 years (36.1, 38.3); No ID & No T2DM: 43.3 years (43.0, 43.7). ^c^The point estimates and confidence intervals for the Chiang’s abridged life table approach represent the additional years expected to live at the start of the 5-year age interval (e.g. at 40 years represents the start of the age interval 40–44 years). The point estimate and confidence intervals at 85 years represent 85+ years. ^d^Additional years expected to live from age 40 years (95% CI): Chiang: ID & T2DM: 32.9 years (26.2, 39.6); ID & No T2DM: 29.9 years (28.2, 31.7); No ID & T2DM: 37.7 years (36.5, 38.9); No ID & No T2DM: 43.3 years (43.0, 43.7)

Figure 2a–d shows the findings using flexible parametric methods for four-way stratification by intellectual disability and T2DM status (Method 1: Fully stratified), with T2DM as an age-varying covariate (Method 2: Partially stratified) and the full model (Method 3: Full model). There are only 761.3 person-years (22 deaths) in the cohort of people with both intellectual disabilities and T2DM and this is reflected in the fully stratified flexible parametric model (Fig. 2a; solid line) where confidence intervals are wide, particularly in the older age groups where numbers are small. For this model and Chiang’s, which also involves stratification, there is an apparent improvement in life expectancy between the ages of 80–84 years. We can also see that the confidence intervals for the group with intellectual disabilities and T2DM are relatively narrow under the Chiang’s approach (Fig. 2a) because the final age group has been combined. This is not ideal given the small sample size (11.9 person years and only 1 death). We can also see that statistical precision is slightly better in the age-varying flexible parametric models for the younger age groups, most noticeably in the first model with the smallest sample size (Fig. 2a) because fewer parameters are required in the models. As before, however, the confidence intervals become wider in the full model (Method 3: Full model) for both intellectual disability samples (Fig. 2a, b; see shaded area). This was even more apparent in the sensitivity analysis where knots were placed at the 0, 25th, 50th, 75th and 100th percentile (Additional file 1: Figure S5).

**Fig. 3:**
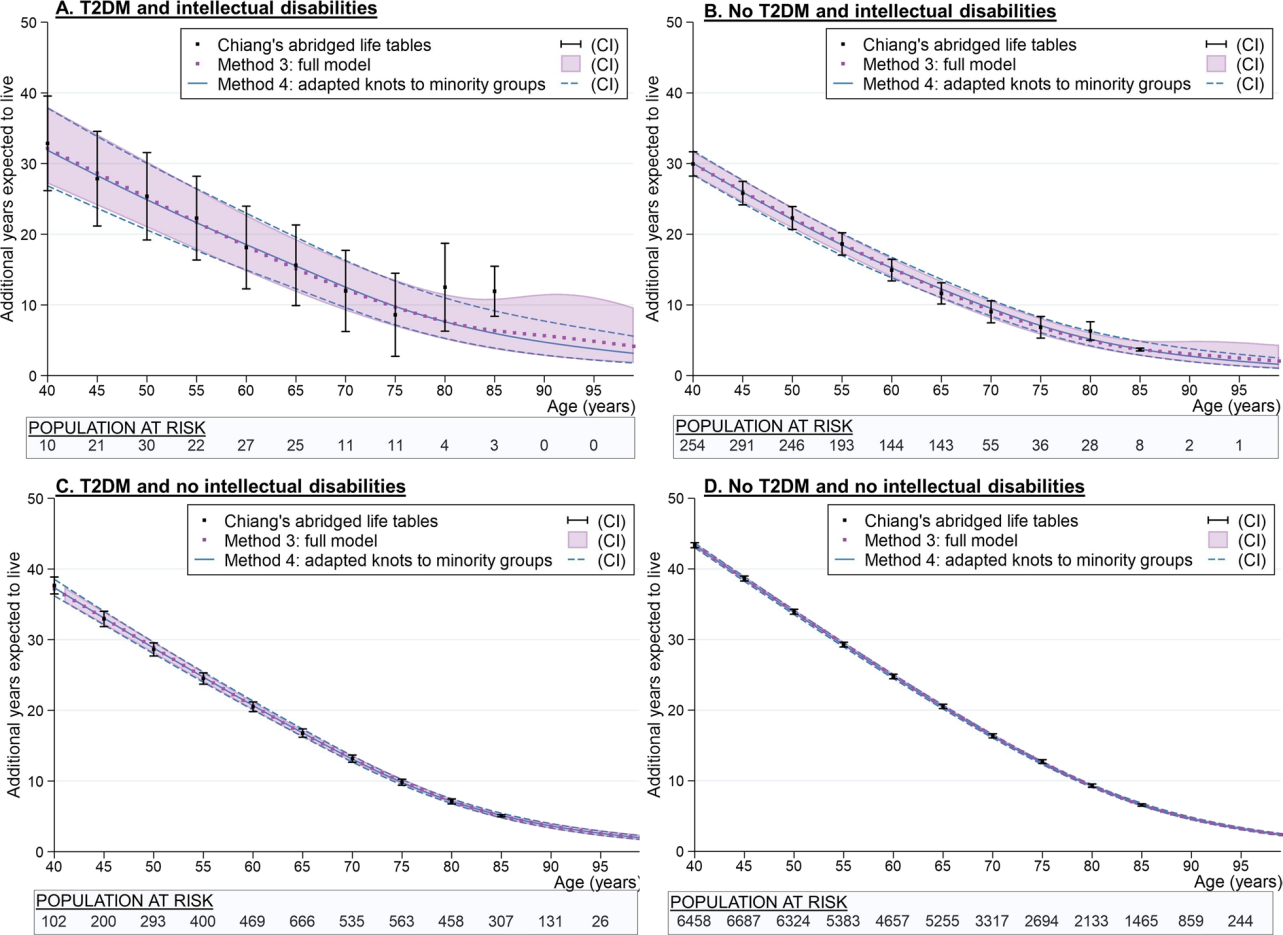
The effect of forcing knots to the minority covariate group in the full model^a^. ^a^Knot placements and expected additional years to live at age 40 are shown in Fig. 2. Additional years expected to live from age 40 years (95% CI): Method 4: Full model with adapted knots (to minority group): ID & T2DM: 31.9 years (26.8, 37.9); ID & No T2DM: 30.1 years (28.4, 31.8); No ID & T2DM: 37.4 years (36.2, 38.5); No ID & No T2DM: 43.3 years (43.0, 43.7)

The placement of knots is, again, a key consideration for these models as they are placed at younger ages in the stratified intellectual disability populations (20.8–81.8 years vs 42.4–87.0 years) because the event distribution is much flatter than the left-skewed distribution observed in both of the groups without intellectual disabilities. By forcing the knots to match the event distribution of the intellectual disability population, we can see that the life expectancy estimates are a closer fit to the actual data (Fig. 3a, b; shaded area). Once knot placements have been considered, the combined flexible parametric methods allow strength to be borrowed from other covariate samples and are, therefore, likely to give a better overall estimate.

Results from bootstrapping the point estimates for life expectancy revealed slightly wider confidence intervals for people with intellectual disabilities and T2DM, particularly in the fully stratified sample, where numbers are very small (see Additional file 1: Figure S1–S3). Given that bootstrapped confidence intervals can be too optimistic for small samples [35], the more conservative confidence intervals produced by the flexible parametric models may be more accurate. Alternative ways to deal with small sample size are considered in the discussion. The percentile-based confidence intervals produced by bootstrapping showed a similar match to the sample as the normal-based confidence intervals and did not allow lower confidence limits to go below zero (see Additional file 1: Figure S4). The simulation study showed a lower risk of bias and less variability using the full model as compared with the stratified model for the 2012 and 2010–2014 sample, but that some bias remained mostly towards improved life expectancy compared to the true (Gompertz) value (see Additional file 1: Figures S7 and S8).

## Discussion

Expectation of life is a simple measure to compare health differences between populations and is often conceptually easier to visualise than other differential mortality comparisons, such as hazard ratios or standardised mortality ratios. This study demonstrates that flexible parametric methods can provide a useful alternative to traditional life table approaches for small populations and have potential applications for both policymakers and researchers.

The limitations of using flexible parametric methods include the need for statistical software to derive the life expectancy estimates as compared with traditional methods that can be done using a spreadsheet. Stata (v16) was used for this work (code shown in the Additional file 1: Box S2), but the methods can be used in other statistical packages such as R (‘*rstpm2’*). We also found that the placement of knots for the restricted cubic splines helped to maximise fit of the data, but we recognise that this could lead to ‘over-fitting’, thereby limiting the model’s ability to predict future life expectancies reliably. We recommend looking at the distribution of deaths at different ages for each stratified covariate prior to conducting life expectancy calculations. We also acknowledge that the predictive ability of the models was less reliable at the tails of the distribution where the population sizes were smaller, but this issue is not restricted to flexible parametric methods and also applies to other life expectancy estimators [7]. In the older age groups, the sample size could be extremely small. While the fully stratified model showed a better fit to the actual data, deaths in a few individuals are unlikely to be a genuine reflection of what is actually happening. Therefore, borrowing strength from larger covariate samples through age-varying models is likely to be more sensible. For very rare covariate patterns, it may be preferable to increase the observation window to two or more years and splitting risk time accordingly, as a trade-off against recency effects.

We also only present findings from the age of 40 years because it was challenging to identify a long-term condition that was present from birth and sufficiently prevalent in people with and without intellectual disabilities. The models for intellectual disabilities from age 10 years closely followed the non-parametric alternative of Chiang’s abridged life table approach (see Additional file 1: Figure S6), but we did not collect information prior to 10 years where differential mortality is likely to have been greater.

We have shown that there are advantages to using flexible parametric methods for life expectancy estimations of sparse data. Parametric models are also appropriate for larger sample sizes by acting in a similar way to Poisson regression, but without the need to split the timescale, which makes predictions easier. These models can also be used in a similar way to propose Bayesian approaches [8] by allowing strength to be borrowed across other regions as covariates with fixed or random effects. Bayesian models have also been shown to be effective in predicting mortality where vital statistics information is missing [36]. In principle, modelling the effects of age interactions as a spline term are simpler models to fit, use fewer parameters and may have benefits in terms of statistical precision and accuracy. However, the reduction in variance using the full model needs to be balanced against the potential increase in bias. Results from the simulation study showed that the full model estimated life expectancy with less bias and variability than the stratified model in this CPRD sample population, but we recommend further work to explore this in different populations. We also observed that the placement of knots can be pulled towards the larger covariate sample so setting knot placements to those of the minority group may be indicated.

## Conclusions

We have shown that flexible parametric methods can be used to calculate life expectancy and have advantages over traditional approaches. The main advantage is that survival curves are smoothed, allowing life expectancy to be calculated by exact age, which is useful for individual-level predictions. The models can also potentially be used to predict different covariate patterns after careful consideration of the proportional effects of the covariates and interactions between them. We recommend further exploration of this novel approach to calculating life expectancy to assess its potential across different settings.

### Supplementary Information

**Additional file 1**. Supplementary material.

## Data Availability

Data for this study were obtained from the Clinical Practice Research Datalink (CPRD), provided by the UK Medicines and Healthcare products Regulatory Agency. The authors’ licence for using these data does not allow sharing of raw data with third parties. Information about access to CPRD data is available here: https://www.cprd.com/research-applications. Researchers should contact the ISAC Secretariat at isac@cprd.com for further details.
