# Supplementary material for: Flexible parametric methods for calculating life expectancy in small populations
Source: Popul Health Metr. 2023 Sep 13;21:13. doi: 10.1186/s12963-023-00313-x (PMC10498577; doi:10.1186/s12963-023-00313-x)
Supplement: Supplementary file 1 — Additional file 1. Supplementary material. [file 12963_2023_313_MOESM1_ESM.docx]

**Flexible parametric methods for calculating life expectancy in small populations**

***Supplementary Material***

**Table S1. Glossary of Terms**

| Flexible parametric models | Flexible parametric methods, first introduced in 2002 [9] and extended further in 2009 [10], provide a parametric alternative to traditional time-to-event analyses by using restricted cubic splines to flexibly and smoothly capture the shape of the baseline log (cumulative) hazard function over time. |
| --- | --- |
| Restricted cubic splines | Restricted cubic splines allow fluctuations and turning points to be captured in survival data. A given covariate is typically transformed to a range of values that are split into ‘knots’, placed within the data range, and connected together using a spline function.  Cubic splines are the most common splines used in survival analyses – they are pieces of polynomial curves (of degree 3) which are joined at knots so that they appear smooth. They are defined as restricted because they are constrained to be linear before the first and after the last knot of the distribution.  For a description of restricted splines in the context of flexible parametric methods, please see Andersson et al. (2013) |
| Interactions | In statistical terms, interactions are where covariates have a non-additive relationship with one another in relation to the outcome. In the context of this study, for example, an interaction between age and T2DM exists if there are age-related differences in mortality between people with and without T2DM. |
| Age-varying covariates | Flexible parametric methods and some other time-to-event analyses allow the effect of covariates to change over time. In the context of the current study, age is modelled as the timescale. Therefore, the relationship between the covariates T2DM and ID are not constrained to be proportional across individual ages. |
| Chiang’s abridged life table approach | Standard life table construction approach from deaths and population data for predefined age intervals, with an open-ended final age interval |
| Stratification | Grouping by certain characteristics of data set, e.g. people with and without T2DM and ID. |

**References**

Andersson TM, Dickman PW, Eloranta S, Lambe M, Lambert PC. Estimating the loss in expectation of life due to cancer using flexible parametric survival models. Statistics in Medicine. 2013;32(30):5286-300.

**Box S1. Formula for calculating life expectancy using flexible parametric methods**

The hazard function, $h(t)$, is defined as the rate of failure between $t$ and $\Delta t$ (i.e. a miniscule time period), conditional on the individual not experiencing the event of interest by time t. The cumulative hazard function, $H(t)$ is the integral of the hazard function, $h(t)$, over the entire distribution of t (i.e. time 0 and time t). The baseline cumulative hazard function is given below.

$$H_{0}\left( t \right)=\int_{o}^{t} h_{0}\left( u \right) du$$

This can be modelled using a parametric form (details below), and the cumulative hazard function is modified to allow for covariate effects using the formula below:

$$H_{i}\left( t \right)=H_{0}\left( t \right)\exp\left( x_{i}\beta\right)$$

Where $H_{0}\left( t \right)$ represents the cumulative hazard function at baseline,$x_{i}\beta$ represents the linear predictor, with $\beta$ representing the log hazard ratios and $x_{\boldsymbol{i}}$ the covariate values for the *i^th^* individual.

The survival function, $S\left( t \right)$*)*, is the probability that an individual will survive beyond time *t*, $\Pr(T>t)$and has a relationship with the cumulative hazard function, as below:

$$S\left( t|\boldsymbol{x}_{\boldsymbol{i}} \right)=exp(-H_{0}\left( t \right)\exp\left( x_{i}\beta\right)=\exp\left( -H_{i}\left( t \right) \right)$$

For flexible parametric methods, the (log) baseline cumulative hazard function is extended using restricted cubic splines to capture the shape of the hazard functions more flexibly (233-235). For example, the whole cumulative hazard function using restricted cubic splines with six knots (i.e. 4df) is defined by:

$$\ln\left[ H\left( t|\boldsymbol{x}_{\boldsymbol{i}} \right) \right]=\gamma_{0}+ \gamma_{1}z_{1i} + \gamma_{2}z_{2i} + \gamma_{3}z_{3i} + \gamma_{4}z_{4i} + \gamma_{5}z_{5i}+ x_{i}\beta$$

Where $\gamma_{0}+\gamma_{1} z_{1i}+\gamma_{2}z_{2i}+\gamma_{3}z_{3i}$ $+ \gamma_{4}z_{4i}$ represents the log cumulative baseline hazard, $\beta$represents the log hazard ratio, $x_{i}$ represents the covariate values for the *i^th^* individual, and *z* represents the spline basis function for $\ln(t)$ for the $i^{th}$ individual (see Royston & Parmar 2002 for mathematical details (227)).

Additional years expected to live from a given age, $a$*,* to a maximum age, $w$*,* can be estimated fitting a flexible parametric model using age as the time scale and integrating under the survival function curve to age $w$, scaled by the survival function (i.e. proportion live) at age *a* using the formula below.

$$\int_{a}^{w} \frac{S\left( t \right) dt}{S\left( a \right)}$$

**Box S2: Stata code for calculating life expectancy**

| * METHOD 1: Fully stratified model  * This is the filename that has split by intellectual disability (ID) and T2DM status (already ‘stset’ – origin=date at 10 years old, enter=date of cohort entry, failure=death, exit=date of death/censoring, ID=patient ID)  * _t=0 represents 10 years old  use "filename", clear    * run flexible parametric models  * ID = intellectual disability status; T2DM = T2DM status  stpm2 if ID==0&T2DM==1&_t>=10, knots(27.5 50 72.5) bknots(5 95) scale(h) knscale(centile)  estimates store ID0T2DM1  stpm2 if ID==0&T2DM==0&_t>=10, knots(27.5 50 72.5) bknots(5 95) scale(h) knscale(centile)  estimates store ID0T2DM0  stpm2 if ID==1&T2DM==1&_t>=10, knots(27.5 50 72.5) bknots(5 95) scale(h) knscale(centile)  estimates store ID1T2DM1  stpm2 if ID==1&T2DM==0&_t>=10, knots(27.5 50 72.5) bknots(5 95) scale(h) knscale(centile)  estimates store ID1T2DM0  quietly {  * note – this is for ages 40–99 years old  forvalues age=30/89 {  capture drop agename  capture drop _LE`age'*  gen age`age'=`age' in 1  estimates restore ID0T2DM1  * Prediction at ID=0, T2DM=1  * The predictions can be captured here to allow for the rare occasion where  * confidence intervals cannot be calculated (shown for this sub-sample only)  capture predictnl _LE_ID0t2dm1`age'=log(predict(rmst tmin(`age') tmax(100)) / predict(surv timevar(age`age'))), ci(_LE_ID0t2dm1_lci`age' _LE_ID0t2dm1_uci`age')  if `c(rc)'==498 {  capture predictnl _LE_ID0t2dm1`age'=predict(rmst tmin(`age') tmax(100)) /predict(surv timevar(age`age'))  gen _LE_ID0t2dm1_lci`age'=.  gen _LE_ID0t2dm1_uci`age'=.  }  else {  replace _LE_ID0t2dm1`age'=exp(_LE_ID0t2dm1`age')  replace _LE_ID0t2dm1_lci`age'=exp(_LE_ID0t2dm1_lci`age')  replace _LE_ID0t2dm1_uci`age'=exp(_LE_ID0t2dm1_uci`age')  }  * Prediction at ID=0, T2DM=0  capture predictnl _LE_ID0t2dm0`age'=log(predict(rmst tmin(`age') tmax(100)) /  predict(surv timevar(age`age'))), ci(_LE_ID0t2dm0_lci`age' _LE_ID0t2dm0_uci`age')  replace _LE_ID0t2dm0`age'=exp(_LE_ID0t2dm0`age')  replace _LE_ID0t2dm0_lci`age'=exp(_LE_ID0t2dm0_lci`age')  replace _LE_ID0t2dm0_uci`age'=exp(_LE_ID0t2dm0_uci`age')  estimates restore ID1T2DM1  * Prediction at ID=1, T2DM=1  capture predictnl _LE_ID1t2dm1`age'=log(predict(rmst tmin(`age') tmax(100)) /  predict(surv timevar(age`age'))), ci(_LE_ID1t2dm1_lci`age' _LE_ID1t2dm1_uci`age')  replace _LE_ID1t2dm1`age'=exp(_LE_ID1t2dm1`age')  replace _LE_ID1t2dm1_lci`age'=exp(_LE_ID1t2dm1_lci`age')  replace _LE_ID1t2dm1_uci`age'=exp(_LE_ID1t2dm1_uci`age')  * Prediction at ID=1, T2DM=0  capture predictnl _LE_ID1t2dm0`age'=log(predict(rmst tmin(`age') tmax(100)) /  predict(surv timevar(age`age'))), ci(_LE_ID1t2dm0_lci`age' _LE_ID1t2dm0_uci`age')  replace _LE_ID1t2dm0`age'=exp(_LE_ID1t2dm0`age')  replace _LE_ID1t2dm0_lci`age'=exp(_LE_ID1t2dm0_lci`age')  replace _LE_ID1t2dm0_uci`age'=exp(_LE_ID1t2dm0_uci`age')  }  } |
| --- |

| * METHOD 2: Partially stratified model  * This is the filename that has split by intellectual disability (ID) and T2DM status (already ‘stset’ – origin=date at 10 years old, enter=date of cohort entry, failure=death, exit=date of death/censoring)  * _t=0 represents 10 years old  use "filename", clear    * run flexible parametric models with T2DM as age-varying covariate  * ID = intellectual disability status; T2DM = T2DM status  stpm2 T2DM if ID==1&_t>=10, knots(27.5 50 72.5) bknots(5 95) scale(h) knscale(centile) dftvc(3) tvc(T2DM)  estimates store ID1T2DM_both  stpm2 T2DM if ID==0&_t>=10, knots(27.5 50 72.5) bknots(5 95) scale(h) knscale(centile) dftvc(3) tvc(T2DM)  estimates store ID0T2DM_both  quietly {  forvalues age=30/89 {  * note – this is for ages 40–99 years old  capture drop agename  capture drop _LE`age'*  gen age`age'=`age' in 1  * Results from ID population  estimates restore ID1T2DM_both  * Prediction at ID=1, T2DM = 1  capture predictnl _LE_ID1_t2dm1both`age'=log(predict(rmst tmin(`age') tmax(100)  at(T2DM 1)) / predict(surv timevar(age`age') at(T2DM 1))), ci(_LE_ID1_t2dm1both_lci`age' _LE_ID1_t2dm1both_uci`age')  replace _LE_ID1_t2dm1both`age' = exp(_LE_ID1_t2dm1both`age')  replace _LE_ID1_t2dm1both_lci`age'= exp(_LE_ID1_t2dm1both_lci`age')  replace _LE_ID1_t2dm1both_uci`age'= exp(_LE_ID1_t2dm1both_uci`age')  * Prediction at ID=1, T2DM=0  capture predictnl _LE_ID1_t2dm0both`age'=log(predict(rmst tmin(`age') tmax(100)  at(T2DM 0)) / predict(surv timevar(age`age') at(T2DM 0))), ci(_LE_ID1_t2dm0both_lci`age' _LE_ID1_t2dm0both_uci`age')  replace _LE_ID1_t2dm0both`age' = exp(_LE_ID1_t2dm0both`age')  replace _LE_ID1_t2dm0both_lci`age'= exp(_LE_ID1_t2dm0both_lci`age')  replace _LE_ID1_t2dm0both_uci`age'= exp(_LE_ID1_t2dm0both_uci`age')  * Results from population without ID  estimates restore ID0T2DM_both  * Prediction at ID=0, T2DM=1  capture predictnl _LE_ID0_t2dm1both`age'=log(predict(rmst tmin(`age') tmax(100)  at(T2DM 1)) / predict(surv timevar(age`age') at(T2DM 1))), ci(_LE_ID0_t2dm1both_lci`age' _LE_ID0_t2dm1both_uci`age')  replace _LE_ID0_t2dm1both`age' = exp(_LE_ID0_t2dm1both`age')  replace _LE_ID0_t2dm1both_lci`age'= exp(_LE_ID0_t2dm1both_lci`age')  replace _LE_ID0_t2dm1both_uci`age'= exp(_LE_ID0_t2dm1both_uci`age')  * Prediction at ID=0, T2DM=0  capture predictnl _LE_ID0_t2dm0both`age'=log(predict(rmst tmin(`age') tmax(100)  at(T2DM 0)) / predict(surv timevar(age`age') at(T2DM 0))), ci(_LE_ID0_t2dm0both_lci`age' _LE_ID0_t2dm0both_uci`age')  replace _LE_ID0_t2dm0both`age' = exp(_LE_ID0_t2dm0both`age')  replace _LE_ID0_t2dm0both_lci`age'= exp(_LE_ID0_t2dm0both_lci`age')  replace _LE_ID0_t2dm0both_uci`age'= exp(_LE_ID0_t2dm0both_uci`age')  }  } |
| --- |

| * METHOD 3: Full model  * This is the filename that has split by intellectual disability (ID) and T2DM status (already ‘stset’ – origin=date at 10 years old, enter=date of cohort entry, failure=death, exit=date of death/censoring)  * _t=0 represents 10 years old  use "filename", clear    * run flexible parametric models with ID and T2DM as age-varying covariates  * ID = intellectual disability status; T2DM = T2DM status  gen int_ID_T2DM=ID*T2DM  stpm2 ID T2DM int_ID_T2DM if _t>=10, knots(27.5 50 72.5) bknots(5 95) scale(h) dftvc(3) tvc(ID T2DM)  quietly {  forvalues age=30/89 {  * note – this is for ages 40–99 years old  capture drop agename  capture drop _LE`age'*  gen age`age'=`age' in 1  * Prediction at ID=1, T2DM = 1  capture predictnl _LE_t2dm1id1`age'=log(predict(rmst tmin(`age') tmax(100) at(T2DM 1 ID 1 int_ID_T2DM 1)) / predict(surv timevar(age`age') at(T2DM 1 ID 1 int_ID_T2DM 1))), ci(_LE_t2dm1id1all_lci`age' _LE_t2dm1id1all_uci`age') replace _LE_t2dm1id1`age'=exp(_LE_t2dm1id1`age')  replace _LE_t2dm1id1all_lci`age'=exp(_LE_t2dm1id1all_lci`age')  replace _LE_t2dm1id1all_uci`age'=exp(_LE_t2dm1id1all_uci`age')  * Prediction at ID=1, T2DM=0  capture predictnl _LE_t2dm1id0`age'=log(predict(rmst tmin(`age') tmax(100) at(T2DM 0 ID 1 int_ID_T2DM 0)) / predict(surv timevar(age`age') at(T2DM 0 ID 1 int_ID_T2DM 0))), ci(_LE_t2dm1id1all_lci`age' _LE_t2dm1id1all_uci`age') replace _LE_t2dm0id1`age'=exp(_LE_t2dm0id1`age')  replace _LE_t2dm0id1all_lci`age'=exp(_LE_t2dm0id1all_lci`age')  replace _LE_t2dm0id1all_uci`age'=exp(_LE_t2dm0id1all_uci`age')  * Prediction at ID=0, T2DM=1  capture predictnl _LE_t2dm1id0`age'=log(predict(rmst tmin(`age') tmax(100) at(T2DM 1 ID 0 int_ID_T2DM 0)) / predict(surv timevar(age`age') at(T2DM 1 ID 0 int_ID_T2DM 0))), ci(_LE_t2dm1id0all_lci`age' _LE_t2dm1id0all_uci`age')  replace _LE_t2dm1id0`age'=exp(_LE_t2dm1id0`age')  replace _LE_t2dm1id0all_lci`age'=exp(_LE_t2dm1id0all_lci`age')  replace _LE_t2dm1id0all_uci`age'=exp(_LE_t2dm1id0all_uci`age')  * Prediction at ID=0, T2DM=0  capture predictnl _LE_t2dm0id0`age'=log(predict(rmst tmin(`age') tmax(100) at(T2DM  0 ID 0 int_ID_T2DM 0)) / predict(surv timevar(age`age') at(T2DM 0 ID 0 int_ID_T2DM 0))), ci(_LE_t2dm0id0all_lci`age' _LE_t2dm0id0all_uci`age')  replace _LE_t2dm0id0`age'=exp(_LE_t2dm0id0`age')  replace _LE_t2dm0id0all_lci`age'=exp(_LE_t2dm0id0all_lci`age')  replace _LE_t2dm0id0all_uci`age'=exp(_LE_t2dm0id0all_uci`age')  }  } |
| --- |

| * METHOD 4: Full model with forced knot placements  * This is the filename that has split by intellectual disability (ID) and T2DM status (already ‘stset’ – origin=date at 10 years old, enter=date of cohort entry, failure=death, exit=date of death/censoring)  * _t=0 represents 10 years old  use "filename", clear    * first run flexible parametric models with T2DM as age-varying covariate  * ID = intellectual disability status; T2DM = T2DM status  stpm2 T2DM if ID==1&_t>=10, knots(27.5 50 72.5) bknots(5 95) scale(h) knscale(centile) dftvc(3) tvc(T2DM)  eret list  * note – the knots can be manually entered or derived function. Here, local variables  * have been created for the knots as below:  * ID_bk1 and ID_bk2 = boundary knots = e(boundary_knots)  * ID_k1, ID_k2 and ID_k3 = knots = e(bhknots)  * ID_tvc1 and ID_tvc2 = knot placements for age-varying effects = e(tvcknots_T2DM)  * Now apply new knots to model  gen int_ID_T2DM=ID*T2DM  stpm2 ID T2DM int_ID_T2DM if _t>=10, knots(`_ID_k1' `_ID_k2' `_ID_k3') bknots(`_ID_bk1' `_ID_bk2') scale(h) knotstvc(T2DM `_ID_tvc1' `_ID_tvc2' ID `_ID_tvc1' `_ID_tvc2' ) tvc(LD T2DM)  * Note – now that the model has been fitted, the below commands are the same as for Method 3  quietly {  forvalues age=30/89 {  * note – this is for ages 40–99 years old  capture drop agename  capture drop _LE`age'*  gen age`age'=`age' in 1  * Prediction at ID=1, T2DM = 1  capture predictnl _LE_t2dm1id1`age'=log(predict(rmst tmin(`age') tmax(100) at(T2DM 1 ID 1 int_ID_T2DM 1)) / predict(surv timevar(age`age') at(T2DM 1 ID 1 int_ID_T2DM 1))), ci(_LE_t2dm1id1all_lci`age' _LE_t2dm1id1all_uci`age') replace _LE_t2dm1id1`age'=exp(_LE_t2dm1id1`age')  replace _LE_t2dm1id1all_lci`age'=exp(_LE_t2dm1id1all_lci`age')  replace _LE_t2dm1id1all_uci`age'=exp(_LE_t2dm1id1all_uci`age')  * Prediction at ID=1, T2DM=0  capture predictnl _LE_t2dm1id0`age'=log(predict(rmst tmin(`age') tmax(100) at(T2DM 0 ID 1 int_ID_T2DM 0)) / predict(surv timevar(age`age') at(T2DM 0 ID 1 int_ID_T2DM 0))), ci(_LE_t2dm1id1all_lci`age' _LE_t2dm1id1all_uci`age') replace _LE_t2dm0id1`age'=exp(_LE_t2dm0id1`age')  replace _LE_t2dm0id1all_lci`age'=exp(_LE_t2dm0id1all_lci`age')  replace _LE_t2dm0id1all_uci`age'=exp(_LE_t2dm0id1all_uci`age')  * Prediction at ID=0, T2DM=1  capture predictnl _LE_t2dm1id0`age'=log(predict(rmst tmin(`age') tmax(100) at(T2DM 1 ID 0 int_ID_T2DM 0)) / predict(surv timevar(age`age') at(T2DM 1 ID 0 int_ID_T2DM 0))), ci(_LE_t2dm1id0all_lci`age' _LE_t2dm1id0all_uci`age')  replace _LE_t2dm1id0`age'=exp(_LE_t2dm1id0`age')  replace _LE_t2dm1id0all_lci`age'=exp(_LE_t2dm1id0all_lci`age')  replace _LE_t2dm1id0all_uci`age'=exp(_LE_t2dm1id0all_uci`age')  * Prediction at ID=0, T2DM=0  capture predictnl _LE_t2dm0id0`age'=log(predict(rmst tmin(`age') tmax(100) at(T2DM  0 ID 0 int_ID_T2DM 0)) / predict(surv timevar(age`age') at(T2DM 0 ID 0 int_ID_T2DM 0))), ci(_LE_t2dm0id0all_lci`age' _LE_t2dm0id0all_uci`age')  replace _LE_t2dm0id0`age'=exp(_LE_t2dm0id0`age')  replace _LE_t2dm0id0all_lci`age'=exp(_LE_t2dm0id0all_lci`age')  replace _LE_t2dm0id0all_uci`age'=exp(_LE_t2dm0id0all_uci`age')  }  } |
| --- |

**Table S2. RECORD^a^ checklist (Reporting of studies conducted using observational routinely-collected data;** [**https://www.record-statement.org/checklist.php**](https://www.record-statement.org/checklist.php)**)**

|  | **Item No.** | **STROBE items** | **Location in manuscript where items are reported** | **RECORD items** | **Location in manuscript where items are reported** |
| --- | --- | --- | --- | --- | --- |
| **Title and Abstract** | | | | | |
|  | 1 | (a) Indicate the study’s design with a commonly used term in the title or the abstract | CPRD mentioned in the abstract | RECORD 1.1: The type of data used should be specified in the title or abstract. When possible, the name of the databases used should be included. | CPRD mentioned in the abstract |
|  |  | (b) Provide in the abstract an informative and balanced summary of what was done and what was found | See abstract | RECORD 1.2: If applicable, the geographic region and timeframe within which the study took place should be reported in the title or abstract. | England and one-year period (2012) both mentioned in the abstract. |
|  |  |  |  | RECORD 1.3: If linkage between databases was conducted for the study, this should be clearly stated in the title or abstract | Linked mortality data referred to in the abstract. |
|  | | | | | |
| Background rationale | 2 | Explain the scientific background and rationale for the investigation being reported | See background |  | |
| Objectives | 3 | State specific objectives, including any prespecified hypotheses | See end of background section |  | |
| **Methods** | | | | | |
| Study Design | 4 | Present key elements of study design early in the paper | See Data sources  (Methods) |  | |
| Setting | 5 | Describe the setting, locations, and relevant dates, including  periods of recruitment, exposure, follow-up, and data collection | See Data sources  (Methods) |  | |
| Participants | 6 | (a) *Cohort study* - Give the eligibility criteria, and the sources and methods of selection of participants. Describe methods of follow-up  *Case-control study* - Give the eligibility criteria, and the sources and methods of case ascertainment and control selection. Give the rationale for the choice of cases and controls  *Cross-sectional study* - Give the eligibility criteria, and the sources and methods of selection of participants | See Data sources and Statistical analyses | RECORD 6.1: The methods of study population selection (such as codes or algorithms used to identify subjects) should be listed in detail. If this is not possible, an explanation should be provided. | All codes are listed in the supplementary material |
|  |  | (b) *Cohort study* For matched studies, give matching criteria and number of exposed and unexposed  *Case-control study* - For matched studies, give matching criteria and the number of controls per case | N/A | RECORD 6.2: Any validation studies of the codes or algorithms used to select the population should be referenced. If validation was conducted for this study and not published elsewhere, detailed methods and results should be provided. | Codes were identified from searches of the literature, free-text searching of Read code descriptions and clinical opinion, referred to in the reference provided but also listed in the supplementary material. |
|  |  |  |  | RECORD 6.3: If the study involved linkage of databases, consider use of a flow diagram or other graphical display to demonstrate the data linkage process, including the number of individuals with linked data at each stage. | Not specified for this article – but available from other publications cited as part of this programme of work. |
| Variables | 7 | Clearly define all outcomes, exposures, predictors, potential confounders, and effect modifiers. Give diagnostic criteria, if applicable. | See Data sources | RECORD 7.1: A complete list of codes and algorithms used to classify exposures, outcomes, confounders, and effect modifiers should be provided. If these cannot be reported, an explanation should be provided. | See supplementary material. |
| Data sources/ measurement | 8 | For each variable of interest, give sources of data and details of methods of assessment (measurement).  Describe comparability of assessment methods if there is more than one group | See Data sources |  | |
| Bias | 9 | Describe any efforts to address potential sources of bias | Analysis adapted to allow intellectual disability to change over time to avoid immortal time bias (see Statistical analyses) |  | |
| Study size | 10 | Explain how the study size was arrived at | Convenience sample of all people with intellectual disabilities and a random sample of people without - See Data sources |  | |
| Quantitative variables | 11 | Explain how quantitative variables were handled in the analyses. If applicable, describe  which groupings were chosen, and why | Binary variables allowed to change over time. Age as the timescale. See Statistical analyses. |  | |
| Statistical methods | 12 | (a) Describe all statistical methods, including those used to control for confounding | See Statistical analyses. |  | |
|  |  | (b) Describe any methods used to examine subgroups and interactions | See Statistical analyses. |  |  |
|  |  | (c) Explain how missing data were addressed | N/A |  |  |
|  |  | (d) *Cohort study* - If applicable, explain how loss to follow-up was addressed  *Case-control study* - If applicable, explain how matching of cases and controls was addressed  *Cross-sectional study* - If applicable, describe analytical methods taking account of sampling strategy | Left and right censoring described in the ‘statistical analyses’ section. Individual-level censored data described in the Introduction. A description of censored data given in the overview (Methods) |  |  |
|  |  | (e) Describe any sensitivity analyses |  |  |  |
| Data access and cleaning methods |  | | | RECORD 12.1: Authors should describe the extent to which the investigators had access to the database population used to create the study population. | This is an established research database that has been quality assessed and checked for internal validity. Relevant papers have been cited in Data sources |
|  |  |  |  | RECORD 12.2: Authors should provide information on the data cleaning methods used in the study. | Relevant codes have been described for this study and how they were used (supplementary material). Data cleaning was minimal because measurements/ results were not used for this analysis. |
| Linkage |  | | | RECORD 12.3: State whether the study included person-level, institutional-level, or other data linkage across two or more databases. The methods of linkage and methods of linkage quality evaluation should be provided. | Person-level linkage was used for this analysis as specified in the methods |
| **Results** | | | | | |
| Participants | 13 | (a) Report the numbers of individuals at each stage of the study (*e.g.*, numbers potentially eligible, examined for eligibility, confirmed eligible, included in the study, completing follow-up, and analysed) | Please see Data sources for initial data selection criteria (from 2000–2019) and subsequent restriction to 2012 only (Statistical analysis) | RECORD 13.1: Describe in detail the selection of the persons included in the study (*i.e.,* study population selection) including filtering based on data quality, data availability and linkage. The selection of included persons can be described in the text and/or by means of the study flow diagram. | See Data sources for initial data selection criteria (from 2000–2019) and subsequent restriction to 2012 only (Statistical analysis). Also Table 2. |
|  |  | (b) Give reasons for non- participation at each stage. | Individuals moving status (intellectual disability and T2DM) and any individuals excluded from different phases of the analyses are described in the footnotes of the tables and figures |  |  |
|  |  | (c) Consider use of a flow diagram | Available on request – flow chart for the wider programme of work (2000 – 2019 cohort is available in previous publications by the same authors) |  |  |
| Descriptive data | 14 | (a) Give characteristics of study participants (*e.g.*, demographic, clinical, social) and information on exposures and potential confounders | See Table 1 |  | |
|  |  | (b) Indicate the number of participants with missing data for each variable of interest | N/A |  |  |
|  |  | (c) *Cohort study* - summarise follow-up time (*e.g.*, average and total amount) | Individuals only followed up for a period of one year (maximum) |  |  |
| Outcome data | 15 | *Cohort study* - Report numbers of outcome events or summary measures over time  *Case-control study* - Report numbers in each exposure category, or summary measures of exposure  *Cross-sectional study* - Report numbers of outcome events or summary measures | See Table 1 |  | |
|  | 16 | (a) Give unadjusted estimates and, if applicable, confounder- adjusted estimates and their precision (e.g., 95% confidence interval). Make clear which confounders were adjusted for and why they were included | Different methods are compared in the manuscript and confidence intervals are provided across all life expectancy estimates. |  | |
|  |  | (b) Report category boundaries when continuous variables were categorized | N/A |  |  |
|  |  | (c) If relevant, consider translating estimates of relative risk into absolute risk for a meaningful time period | Life expectancy estimates provide absolute life years lost, although this was not the prime focus of this methodological work. |  |  |
| Other analyses | 17 | Report other analyses done— e.g., analyses of subgroups and interactions, and sensitivity analyses | Sensitivity analyses are described in the supplementary material. |  | |
| **Discussion** | | | | | |
| Key results | 18 | Summarise key results with  reference to study objectives | See first paragraph of Discussion |  | |
| Limitations | 19 | Discuss limitations of the study, taking into account sources of potential bias or imprecision.  Discuss both direction and magnitude of any potential bias | See second paragraph of Discussion. | RECORD 19.1: Discuss the implications of using data that were not created or collected to answer the specific research question(s). Include discussion of misclassification bias, unmeasured confounding, missing data, and changing eligibility over time, as they pertain to the study being  reported. | The data used for this study were not collected by the researchers but the data have been quality assessed and checked for internal validity as part of the CPRD’s quality control procedures. Relevant papers have been cited in Data sources. The issues of immortal time bias have been addressed by treating the exposure as an age-dependent covariate. This methodological work does not seek to explore the relationship between life expectancy and intellectual disability – rather, to explore flexible parametric methods as an approach to calculating life expectancy and how knot placements may be adapted for smaller populations. |
| Interpretation | 20 | Give a cautious overall interpretation of results considering objectives, limitations, multiplicity of analyses, results from similar studies, and other relevant evidence | See Discussion |  | |
| Generalisability | 21 | Discuss the generalisability (external validity) of the study results | See last paragraph of Discussion. |  | |
| **Other Information** | | | | | |
| Funding | 22 | Give the source of funding and the role of the funders for the present study and, if applicable, for the original study on which the present article is based | See Competing Interests & Acknowledgements |  | |
| Accessibility of protocol, raw data, and programming  code |  | | | RECORD 22.1: Authors should provide information on how to access any supplemental information such as the study protocol, raw data, or programming code. | The CPRD’s ISAC study protocol ID is listed and available to view on-line (**Data Sources**). Raw data are not available and an explanation is given for this in the **Data sharing statement**. |

Checklist is protected under Creative Commons Attribution ([CC BY](http://creativecommons.org/licenses/by/4.0/)) licence.

^a^ Reference: Benchimol EI, Smeeth L, Guttmann A, Harron K, Moher D, Petersen I, Sørensen HT, von Elm E, Langan SM, the RECORD Working Committee. The REporting of studies Conducted using Observational Routinely-collected health Data (RECORD) Statement. *PLoS Medicine* 2015; 2(10): e1001885. https://doi.org/10.1371/journal.pmed.1001885

**Table S3. Diagnostic and classification codes for intellectual disabilities and T2DM**

| **Primary care Read codes for intellectual disability diagnoses**  *From clinical, referral or tests data* | |
| --- | --- |
| **Read Code** | **Read Code Description** |
| C31yX00 | Disorder of glycoprotein metabolism, unspecified |
| C372000 | Hypoxanthine-guanine-phosphoribosyltransferase deficiency |
| C372011 | Lesch - Nyhan syndrome |
| C372300 | Lesch-Nyhan syndrome |
| C372z00 | Other disorder of purine or pyrimidine metabolism NOS |
| E141000 | Active disintegrative psychoses |
| E141100 | Residual disintegrative psychoses |
| E141z00 | Disintegrative psychosis NOS |
| Eu70000 | [X]Mld mental retard with statement no or min impairm behav |
| Eu70100 | [X]Mld mental retard sig impairment behav req attent/treatmt |
| Eu70y00 | [X]Mild mental retardation, other impairments of behaviour |
| Eu70z00 | [X]Mild mental retardation without mention impairment behav |
| Eu71000 | [X]Mod mental retard with statement no or min impairm behav |
| Eu71100 | [X]Mod mental retard sig impairment behav req attent/treatmt |
| Eu71y00 | [X]Mod retard oth behav impair |
| Eu71z00 | [X]Mod mental retardation without mention impairment behav |
| Eu72000 | [X]Sev mental retard with statement no or min impairm behav |
| Eu72100 | [X]Sev mental retard sig impairment behav req attent/treatmt |
| Eu72y00 | [X]Severe mental retardation, other impairments of behaviour |
| Eu72z00 | [X]Sev mental retardation without mention impairment behav |
| Eu73000 | [X]Profound ment retrd wth statement no or min impairm behav |
| Eu73100 | [X]Profound ment retard sig impairmnt behav req attent/treat |
| Eu73y00 | [X]Profound mental retardation, other impairments of behavr |
| Eu73z00 | [X]Prfnd mental retardation without mention impairment behav |
| Eu7y000 | [X]Oth mental retard with statement no or min impairm behav |
| Eu7y100 | [X]Oth mental retard sig impairment behav req attent/treatmt |
| Eu7yy00 | [X]Other mental retardation, other impairments of behaviour |
| Eu7yz00 | [X]Other mental retardation without mention impairment behav |
| Eu7z000 | [X]Unsp mental retard with statement no or min impairm behav |
| Eu7z100 | [X]Unsp mentl retard sig impairment behav req attent/treatmt |
| Eu7zy00 | [X]Unspecified mental retardatn, other impairments of behav |
| Eu7zz00 | [X]Unsp mental retardation without mention impairment behav |
| Eu81400 | [X]Moderate learning disability |
| Eu81500 | [X]Severe learning disability |
| Eu81600 | [X]Mild learning disability |
| Eu81700 | [X]Profound learning disability |
| Eu81800 | [X]Specific learning disability |
| Eu81z00 | [X]Developmental disorder of scholastic skills, unspecified |
| Eu81z11 | [X]Learning disability NOS |
| Eu81z12 | [X]Learning disorder NOS |
| Eu81z13 | [X]Learn acquisition disab NOS |
| Eu84112 | [X]Mental retardation with autistic features |
| Eu84200 | [X]Rett's syndrome |
| Eu84300 | [X]Other childhood disintegrative disorder |
| Eu84311 | [X]Dementia infantalis |
| Eu84312 | [X]Disintegrative psychosis |
| Eu84313 | [X]Heller's syndrome |
| Eu84400 | [X]Overactive disorder assoc mental retard/stereotype movts |
| P22yz00 | Other reduction deformity of brain NOS |
| PJ33100 | Deletion of long arm of chromosome 18 |
| PJ33111 | 18p- syndrome |
| PJ33200 | Deletion of short arm of chromosome 18 |
| PJ33211 | 18q- syndrome |
| PJ33300 | Smith-Magenis syndrome |
| PJ33400 | Jacobsen syndrome |
| PJ33500 | Greig cephalopolysyndactyly syndrome |
| PJ33700 | 3p deletion syndrome |
| PJ33800 | Chromosome 4q deletion syndrome |
| PJ33900 | Langer-Giedion syndrome |
| PJ33A00 | Kleefstra syndrome |
| PJ50000 | Trisomy 6 |
| PJ50100 | Trisomy 7 |
| PJ50200 | Trisomy 8 |
| PJ50300 | Trisomy 9 |
| PJ50400 | Trisomy 10 |
| PJ50500 | Trisomy 11 |
| PJ50600 | Trisomy 12 |
| PJ50700 | Other trisomy C syndromes |
| PJ50800 | Trisomy 22 |
| PJ50w00 | Whole chromosome trisomy, meitotic nondisjunction |
| PJ50x00 | Whole chromosome trisomy, mosaicism |
| PJ50x11 | Whole chromosome trisomy, mitotic nondisjunction |
| PJ50y00 | Other specified whole chromosome trisomy syndrome |
| PJ50z00 | Whole chromosome trisomy syndrome NOS |
| PJ51000 | Major partial trisomy |
| PJ51100 | Minor partial trisomy |
| PJ51200 | 10q partial trisomy syndrome |
| PJ51300 | Trisomy 4p syndrome |
| PJ51400 | Trisomy 9p syndrome |
| PJ51500 | 15q partial trisomy syndrome |
| PJ51z00 | Partial trisomy syndrome NOS |
| PJ52300 | Triploidy |
| PJ52400 | Polyploidy |
| PJ52z00 | Trisomy of autosomes NEC NOS |
| PJyy200 | Fragile X chromosome |
| PJyy400 | Fragile X syndrome |
| PKy6100 | Cockayne syndrome |
| PKy9300 | Prader - Willi syndrome |
| PKyz.11 | Cockayne's syndrome |
| PKyz511 | Angelman syndrome |
| PKyz700 | Angelman's syndrome |
| Pyu0200 | [X]Other reduction deformities of brain |
| Pyu0300 | [X]Other specified congenital malformations of brain |
| PyuA000 | [X]Oth specif trisomies & partial trisomies of autosomes |
| R034y11 | [D]Global retardation |
| ZL1B500 | Under care of psychiatrist for mental handicap |
| 918e.00 | On learning disability register |
| C031.00 | Goitrous cretin |
| C0A..00 | Congenital iodine deficiency syndrome |
| C0A0.00 | Congenital iodine-deficiency syndrome, neurological type |
| C0A1.00 | Congenital iodine-deficiency syndrome, myxoedematous type |
| C301.00 | Phenylketonuria |
| C372.00 | Other disorders of purine and pyrimidine metabolism |
| C377.00 | Disorders of glycoprotein metabolism |
| E141.00 | Disintegrative psychosis |
| E3..00 | Mental retardation |
| E30..00 | Mild mental retardation, IQ in range 50-70 |
| E31..00 | Other specified mental retardation |
| E310.00 | Moderate mental retardation, IQ in range 35-49 |
| E311.00 | Severe mental retardation, IQ in range 20-34 |
| E312.00 | Profound mental retardation with IQ less than 20 |
| E31z.00 | Other specified mental retardation NOS |
| E3y..00 | Other specified mental retardation |
| E3z..00 | Mental retardation NOS |
| Eu7..00 | [X]Mental retardation |
| Eu70.00 | [X]Mild mental retardation |
| Eu71.00 | [X]Moderate mental retardation |
| Eu72.00 | [X]Severe mental retardation |
| Eu73.00 | [X]Profound mental retardation |
| Eu7y.00 | [X]Other mental retardation |
| Eu7z.00 | [X]Unspecified mental retardation |
| P01..00 | Craniorachischisis |
| P02..00 | Iniencephaly |
| P22..00 | Reduction deformities of brain |
| P224.00 | Arhinencephaly |
| P225.00 | Holoprosencephaly |
| P22y.00 | Other specified reduction deformities of brain |
| P22z.00 | Reduction deformities of brain NOS |
| PJ0..00 | Down's syndrome - trisomy 21 |
| PJ00.00 | Trisomy 21, meiotic nondisjunction |
| PJ02.00 | Trisomy 21, translocation |
| PJ0z.00 | Down's syndrome NOS |
| PJ1..00 | Patau's syndrome - trisomy 13 |
| PJ10.00 | Trisomy 13, meiotic nondisjunction |
| PJ11.00 | Trisomy 13, mosaicism |
| PJ12.00 | Trisomy 13, translocation |
| PJ1z.00 | Patau's syndrome NOS |
| PJ2..00 | Edward's syndrome - trisomy 18 |
| PJ20.00 | Trisomy 18, meiotic nondisjunction |
| PJ21.00 | Trisomy 18, mosaicism |
| PJ22.00 | Trisomy 18, translocation |
| PJ2z.00 | Edward's syndrome NOS |
| PJ30.00 | Antimongolism syndrome |
| PJ31.00 | Cri-du-chat syndrome |
| PJ32.00 | Deletion of short arm of chromosome 4 |
| PJ3z.00 | Monosomies and deletions from the autosomes NOS |
| PJ50.00 | Whole chromosome trisomy syndromes |
| PJ51.00 | Partial trisomy syndromes |
| PJ52.00 | Trisomies of autosomes NEC |
| PJ9..00 | Mowat-Wilson syndrome |
| PK5..00 | Tuberous sclerosis |
| PKy4.00 | William syndrome |
| ZS34.00 | Developmental disorder of scholastic skill |
| E3...00 | Mental retardation |
| C03..11 | Cretinism |
| C372.11 | Lesch - Nyhan syndrome |
| E141.11 | Heller's syndrome |
| E30..11 | Educationally subnormal |
| E310.11 | Imbecile |
| E312.11 | Idiocy |
| Eu70.11 | [X]Feeble-mindedness |
| Eu71.11 | [X]Moderate mental subnormality |
| Eu72.11 | [X]Severe mental subnormality |
| Eu73.11 | [X]Profound mental subnormality |
| Eu7z.11 | [X]Mental deficiency NOS |
| PJ0..11 | Mongolism |
| PJ01.11 | Trisomy 21, mitotic nondisjunction |
| PJ02.11 | Partial trisomy 21 in Down's syndrome |
| PJ0z.11 | Trisomy 21 NOS |
| PJ11.11 | Trisomy 13, mitotic nondisjunction |
| PJ12.11 | Partial trisomy 13 in Patau's syndrome |
| PJ1z.11 | Trisomy 13 NOS |
| PJ21.11 | Trisomy 18, mitotic nondisjunction |
| PJ22.11 | Partial trisomy 18 in Edward's syndrome |
| PJ2z.11 | TRISOMY 18 NOS |
| PJ30.11 | Deletion of long arm of chromosome 21 |
| PJ31.11 | Deletion of short arm of chromosome 5 |
| PJ32.11 | Wolff - Hirschorn syndrome |
| PKy0.11 | Prader-Willi Syndrome |
| ZS34.11 | Learning disability |
| C03z.12 | Cretinism |
| E30..12 | Feeble-minded |
| Eu70.12 | [X]Mild mental subnormality |
| Eu7z.12 | [X]Mental subnormality NOS |
| PJ0..12 | Trisomy 21 |
| PKy0.12 | Prader-Willi syndrome |
| E30..13 | Moron |
| PJ0..13 | Trisomy 22 |
| 8Ce6.00 | Preferred place of care - learning disability unit |
| 9HB..00 | Learning disabilities administration status |
| 9HB0.00 | Learning disabilities health action plan declined |
| 9HB1.00 | Learning disabilities health action plan offered |
| 9HB2.00 | Learning disabilities health action plan reviewed |
| 9HB3.00 | Learning disabilities health assessment |
| 9HB4.00 | Learning disabilities health action plan completed |
| 9HB5.00 | Learning disabilities annual health assessment |
| 9HB6.00 | Learning disabilities annual health assessment declined |
| 9HB7.00 | Did not attend learning disabilities annual health assessmnt |
| 9hL..00 | Exception reporting: learning disability quality indicators |
| 9hL0.00 | Exc learn disability quality indicators: informed dissent |
| 9hL1.00 | Exc learn disability quality indicators: patient unsuitable |
| 9mA..00 | Learning disability annual health check invitation |
| 9mA0.00 | Learning disability annual health check verbal invitation |
| 9mA1.00 | Learning disability annual health check telephone invitation |
| 9mA2.00 | Learning disability annual health check letter invitation |
| 9mA2000 | Learning disability annual health check invtation 1st letter |
| 9mA2100 | Learning disability annual health check invtation 2nd letter |
| 9mA2200 | Learning disability annual health check invtation 3rd letter |
| 9Nh4.00 | Under care of community learning disability team |
| 9HB6.11 | Learning disabilities annual health check declined |
| 9HB7.11 | Did not attend learning disabilities annual health check |
| 13Z3.00 | Low I.Q. |
| 69DB.00 | Learning disability health examination |
| 94Z9.00 | Preferred place of death: learning disability unit |
| 6664 | Mental handicap problem |
| **Primary care Read codes for T2DM**  *From clinical, referral or tests data* | |
| **Read Code** | **Read Code Description** |
| 66A3.00 | Diabetic on diet only |
| 66A4.00 | Diabetic on oral treatment |
| 66Ao.00 | Diabetes type 2 review |
| 66At.00 | Diabetic dietary review |
| 66At100 | Type II diabetic dietary review |
| 66At111 | Type 2 diabetic dietary review |
| 66AV.00 | Diabetic on insulin and oral treatment |
| 66AY.00 | Diabetic diet - good compliance |
| 9OLE.00 | Attended DESMOND structured programme |
| 9OLG.00 | Attended XPERT diabetes structured education programme |
| 9OLK.00 | DESMOND diabetes structured education programme completed |
| 9OLL.00 | XPERT diabetes structured education programme completed |
| C100100 | Diabetes mellitus, adult onset, no mention of complication |
| C100111 | Maturity onset diabetes |
| C100112 | Non-insulin dependent diabetes mellitus |
| C101100 | Diabetes mellitus, adult onset, with ketoacidosis |
| C102100 | Diabetes mellitus, adult onset, with hyperosmolar coma |
| C103100 | Diabetes mellitus, adult onset, with ketoacidotic coma |
| C105100 | Diabetes mellitus, adult onset, + ophthalmic manifestation |
| C106100 | Diabetes mellitus, adult onset, + neurological manifestation |
| C107.00 | Diabetes mellitus with peripheral circulatory disorder |
| C107100 | Diabetes mellitus, adult, + peripheral circulatory disorder |
| C107200 | Diabetes mellitus, adult with gangrene |
| C107400 | NIDDM with peripheral circulatory disorder |
| C109.00 | Non-insulin dependent diabetes mellitus |
| C109.11 | NIDDM - Non-insulin dependent diabetes mellitus |
| C109.12 | Type 2 diabetes mellitus |
| C109.13 | Type II diabetes mellitus |
| C109000 | Non-insulin-dependent diabetes mellitus with renal comps |
| C109011 | Type II diabetes mellitus with renal complications |
| C109012 | Type 2 diabetes mellitus with renal complications |
| C109100 | Non-insulin-dependent diabetes mellitus with ophthalm comps |
| C109111 | Type II diabetes mellitus with ophthalmic complications |
| C109112 | Type 2 diabetes mellitus with ophthalmic complications |
| C109200 | Non-insulin-dependent diabetes mellitus with neuro comps |
| C109211 | Type II diabetes mellitus with neurological complications |
| C109212 | Type 2 diabetes mellitus with neurological complications |
| C109300 | Non-insulin-dependent diabetes mellitus with multiple comps |
| C109312 | Type 2 diabetes mellitus with multiple complications |
| C109400 | Non-insulin dependent diabetes mellitus with ulcer |
| C109411 | Type II diabetes mellitus with ulcer |
| C109412 | Type 2 diabetes mellitus with ulcer |
| C109500 | Non-insulin dependent diabetes mellitus with gangrene |
| C109511 | Type II diabetes mellitus with gangrene |
| C109512 | Type 2 diabetes mellitus with gangrene |
| C109600 | Non-insulin-dependent diabetes mellitus with retinopathy |
| C109611 | Type II diabetes mellitus with retinopathy |
| C109612 | Type 2 diabetes mellitus with retinopathy |
| C109700 | Non-insulin dependent diabetes mellitus - poor control |
| C109711 | Type II diabetes mellitus - poor control |
| C109712 | Type 2 diabetes mellitus - poor control |
| C109900 | Non-insulin-dependent diabetes mellitus without complication |
| C109911 | Type II diabetes mellitus without complication |
| C109912 | Type 2 diabetes mellitus without complication |
| C109A00 | Non-insulin dependent diabetes mellitus with mononeuropathy |
| C109A11 | Type II diabetes mellitus with mononeuropathy |
| C109B00 | Non-insulin dependent diabetes mellitus with polyneuropathy |
| C109B11 | Type II diabetes mellitus with polyneuropathy |
| C109B12 | Type 2 diabetes mellitus with polyneuropathy |
| C109C00 | Non-insulin dependent diabetes mellitus with nephropathy |
| C109C11 | Type II diabetes mellitus with nephropathy |
| C109C12 | Type 2 diabetes mellitus with nephropathy |
| C109D00 | Non-insulin dependent diabetes mellitus with hypoglyca coma |
| C109D11 | Type II diabetes mellitus with hypoglycaemic coma |
| C109D12 | Type 2 diabetes mellitus with hypoglycaemic coma |
| C109E00 | Non-insulin depend diabetes mellitus with diabetic cataract |
| C109E11 | Type II diabetes mellitus with diabetic cataract |
| C109E12 | Type 2 diabetes mellitus with diabetic cataract |
| C109F00 | Non-insulin-dependent d m with peripheral angiopath |
| C109F11 | Type II diabetes mellitus with peripheral angiopathy |
| C109F12 | Type 2 diabetes mellitus with peripheral angiopathy |
| C109G00 | Non-insulin dependent diabetes mellitus with arthropathy |
| C109G11 | Type II diabetes mellitus with arthropathy |
| C109G12 | Type 2 diabetes mellitus with arthropathy |
| C109H00 | Non-insulin dependent d m with neuropathic arthropathy |
| C109H11 | Type II diabetes mellitus with neuropathic arthropathy |
| C109H12 | Type 2 diabetes mellitus with neuropathic arthropathy |
| C109J00 | Insulin treated Type 2 diabetes mellitus |
| C109J11 | Insulin treated non-insulin dependent diabetes mellitus |
| C109J12 | Insulin treated Type II diabetes mellitus |
| C109K00 | Hyperosmolar non-ketotic state in type 2 diabetes mellitus |
| C10D.00 | Diabetes mellitus autosomal dominant type 2 |
| C10D.11 | Maturity onset diabetes in youth type 2 |
| C10ER00 | Latent autoimmune diabetes mellitus in adult |
| C10F.00 | Type 2 diabetes mellitus |
| C10F.11 | Type II diabetes mellitus |
| C10F000 | Type 2 diabetes mellitus with renal complications |
| C10F011 | Type II diabetes mellitus with renal complications |
| C10F100 | Type 2 diabetes mellitus with ophthalmic complications |
| C10F111 | Type II diabetes mellitus with ophthalmic complications |
| C10F200 | Type 2 diabetes mellitus with neurological complications |
| C10F211 | Type II diabetes mellitus with neurological complications |
| C10F300 | Type 2 diabetes mellitus with multiple complications |
| C10F311 | Type II diabetes mellitus with multiple complications |
| C10F400 | Type 2 diabetes mellitus with ulcer |
| C10F411 | Type II diabetes mellitus with ulcer |
| C10F500 | Type 2 diabetes mellitus with gangrene |
| C10F511 | Type II diabetes mellitus with gangrene |
| C10F600 | Type 2 diabetes mellitus with retinopathy |
| C10F611 | Type II diabetes mellitus with retinopathy |
| C10F700 | Type 2 diabetes mellitus - poor control |
| C10F711 | Type II diabetes mellitus - poor control |
| C10F900 | Type 2 diabetes mellitus without complication |
| C10F911 | Type II diabetes mellitus without complication |
| C10FA00 | Type 2 diabetes mellitus with mononeuropathy |
| C10FA11 | Type II diabetes mellitus with mononeuropathy |
| C10FB00 | Type 2 diabetes mellitus with polyneuropathy |
| C10FB11 | Type II diabetes mellitus with polyneuropathy |
| C10FC00 | Type 2 diabetes mellitus with nephropathy |
| C10FC11 | Type II diabetes mellitus with nephropathy |
| C10FD00 | Type 2 diabetes mellitus with hypoglycaemic coma |
| C10FD11 | Type II diabetes mellitus with hypoglycaemic coma |
| C10FE00 | Type 2 diabetes mellitus with diabetic cataract |
| C10FE11 | Type II diabetes mellitus with diabetic cataract |
| C10FF00 | Type 2 diabetes mellitus with peripheral angiopathy |
| C10FF11 | Type II diabetes mellitus with peripheral angiopathy |
| C10FG00 | Type 2 diabetes mellitus with arthropathy |
| C10FG11 | Type II diabetes mellitus with arthropathy |
| C10FH00 | Type 2 diabetes mellitus with neuropathic arthropathy |
| C10FH11 | Type II diabetes mellitus with neuropathic arthropathy |
| C10FJ00 | Insulin treated Type 2 diabetes mellitus |
| C10FJ11 | Insulin treated Type II diabetes mellitus |
| C10FK00 | Hyperosmolar non-ketotic state in type 2 diabetes mellitus |
| C10FK11 | Hyperosmolar non-ketotic state in type II diabetes mellitus |
| C10FL00 | Type 2 diabetes mellitus with persistent proteinuria |
| C10FL11 | Type II diabetes mellitus with persistent proteinuria |
| C10FM00 | Type 2 diabetes mellitus with persistent microalbuminuria |
| C10FM11 | Type II diabetes mellitus with persistent microalbuminuria |
| C10FN00 | Type 2 diabetes mellitus with ketoacidosis |
| C10FN11 | Type II diabetes mellitus with ketoacidosis |
| C10FP00 | Type 2 diabetes mellitus with ketoacidotic coma |
| C10FP11 | Type II diabetes mellitus with ketoacidotic coma |
| C10FQ00 | Type 2 diabetes mellitus with exudative maculopathy |
| C10FQ11 | Type II diabetes mellitus with exudative maculopathy |
| C10FR00 | Type 2 diabetes mellitus with gastroparesis |
| C10P100 | Type II diabetes mellitus in remission |
| C10P111 | Type 2 diabetes mellitus in remission |
| C10y100 | Diabetes mellitus, adult, + other specified manifestation |
| L180600 | Pre-existing diabetes mellitus, non-insulin-dependent |
| ZC2CA00 | Dietary advice for type II diabetes |
| **Hospital ICD-10 (version 10 of International Classification of Diseases) codes for T2DM** | |
| **ICD-10 Code** | **ICD-10 code Description** |
| E11 | Type 2 diabetes mellitus |

T2DM: Type 2 diabetes mellitus

**Figure S1. Comparison between bootstrapped confidence intervals and log-transformed confidence intervals at ages 80–99 years in people with intellectual disabilities**

**Method 1: fully stratified**

T2DM: Type 2 diabetes; CI: confidence intervals

The model included *n*=46 individuals with intellectual disabilities aged 80+ years and T2DM [*n*=1 aged 99+ years] and *n*=158 with intellectual disabilities without T2DM [none aged 99+ years]

**Figure S2. Comparison between bootstrapped confidence intervals and log-transformed confidence intervals at ages 80–99 years in people with intellectual disabilities**

**Method 2: partially stratified**

T2DM: Type 2 diabetes; CI: confidence intervals

The model included *n*=46 individuals with intellectual disabilities aged 80+ years and T2DM [*n*=1 aged 99+ years] and *n*=158 with intellectual disabilities without T2DM aged 80+ years [none aged 99+ years]

**Figure S3. Comparison between bootstrapped confidence intervals and log-transformed confidence intervals at ages 80–99 years in people with intellectual disabilities**

**Method 3: full model**

T2DM: Type 2 diabetes; CI: confidence intervals

The model included *n*=46 individuals with intellectual disabilities and T2DM [*n*=1 aged 99+ years] and *n*=158 with intellectual disabilities without T2DM [none aged 99+ years]. It also borrowed power from the cohort without intellectual disabilities (*n*= 26,619 aged 80+ years; *n*=233 aged 99+ years]

**Figure S4. Comparison between bootstrapped confidence intervals (percentile and normal) and flexible parametric methods at ages 95–105 in people without intellectual disabilities**

CI: confidence intervals

**Figure S5. Comparison between combined flexible parametric model (default [Stata] and Harrell’s knot placements) and Chiang’s abridged life table approach for life expectancy from age 40 years**

**
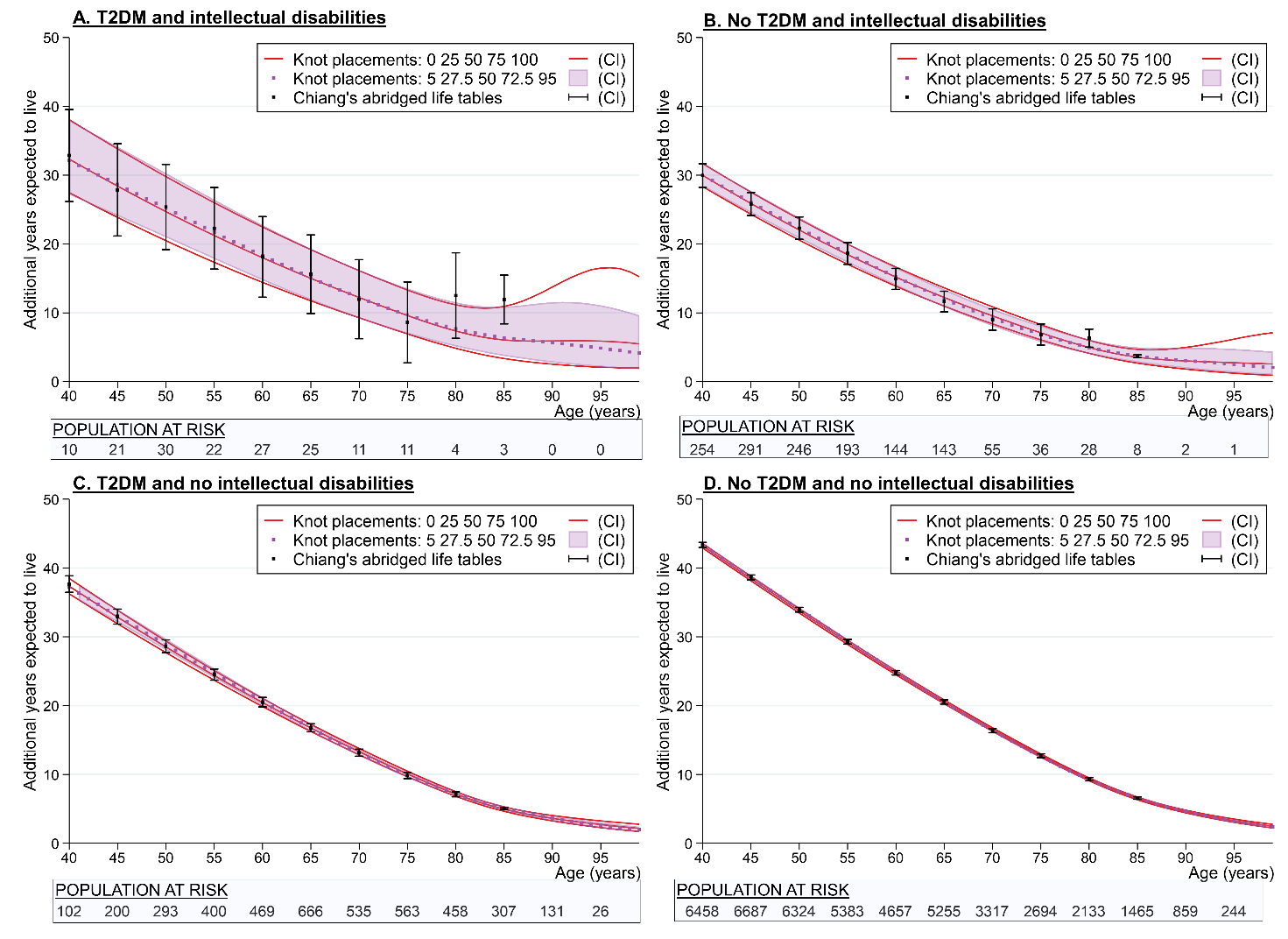
**

**Figure S6. Comparison between flexible parametric model and Chiang’s abridged life table approach for life expectancy from age 10 years**

**Figure S7. Comparison between simulated stratified and full flexible parametric models and true (Gompertz) value: 2012 data**

**
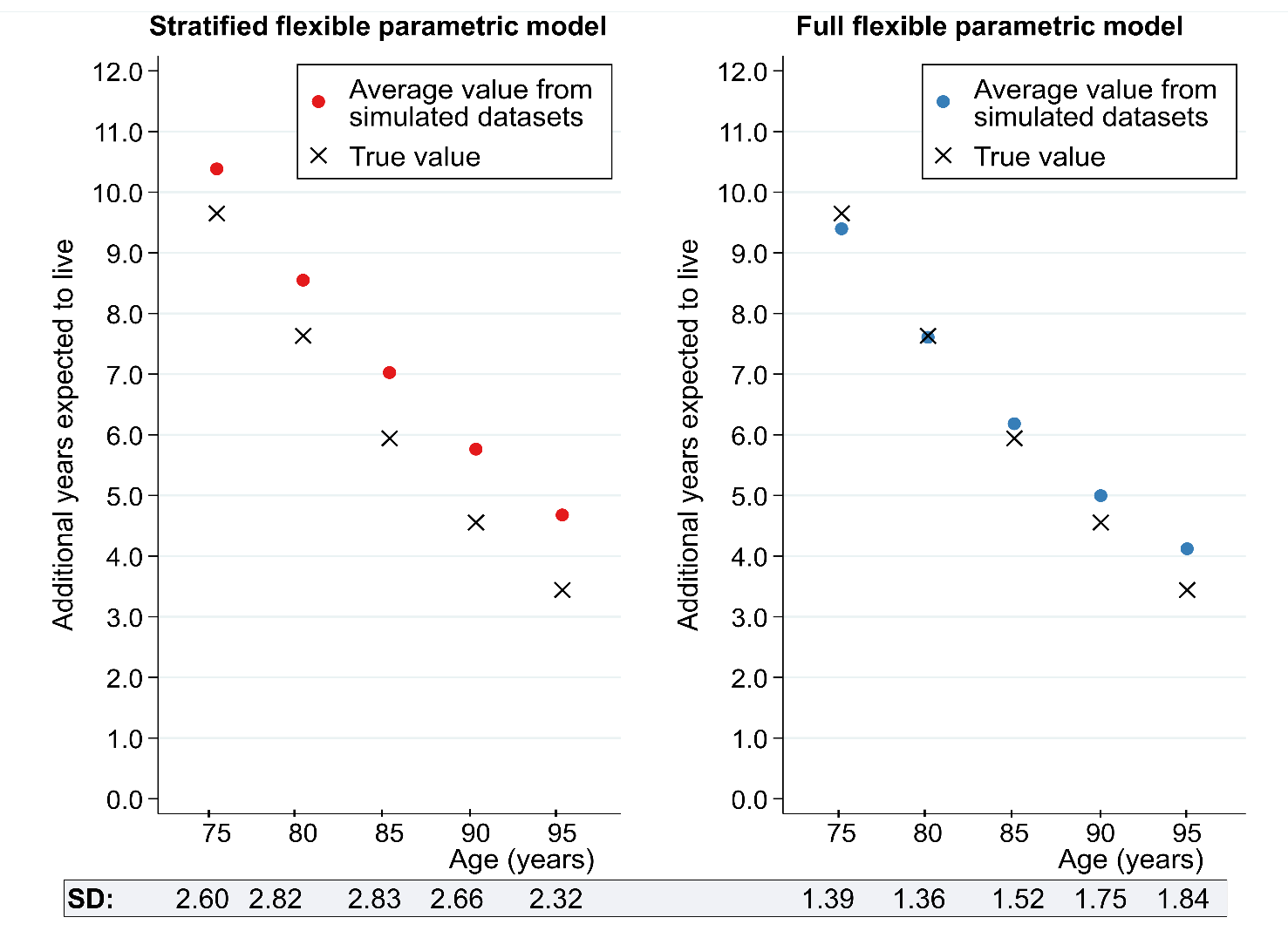
**

* Data that had the same covariate pattern were repeatedly recreated (n=1000 iterations; only models that both converged were included [n=874 iterations]) and the stratified and full models compared. The results are shown for the smallest group (i.e. intellectual disability and T2DM) for 75+years

**Figure S8. Comparison between simulated stratified and full flexible parametric models and true (Gompertz) value: 2010–2014 data***

**
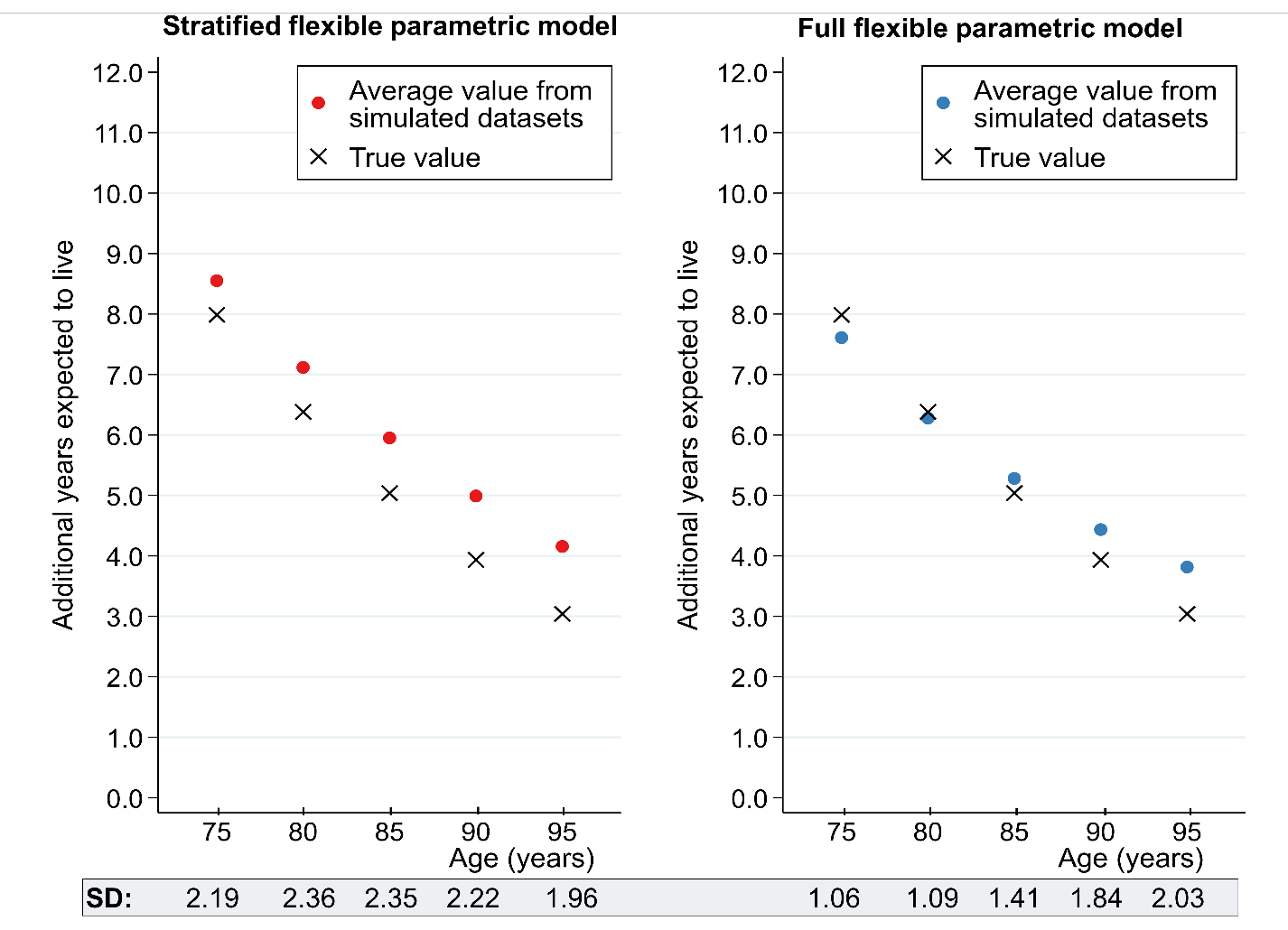
**

* using extended data set from 1 Jan 2010 to 31 Dec 2014 to better capture the true shape of survival in the sample population. Data that had the same covariate pattern were repeatedly recreated (n=1000 iterations; only models that both converged were included [n=927]) and the stratified and full models compared. The results are shown for the smallest group (i.e. intellectual disability and T2DM) for 75+years
